# Healthcare utilization and maternal and child mortality during the COVID-19 pandemic in 18 low- and middle-income countries: An interrupted time-series analysis with mathematical modeling of administrative data

**DOI:** 10.1371/journal.pmed.1004070

**Published:** 2022-08-30

**Authors:** Tashrik Ahmed, Timothy Roberton, Petra Vergeer, Peter M. Hansen, Michael A. Peters, Anthony Adofo Ofosu, Charles Mwansambo, Charles Nzelu, Chea Sanford Wesseh, Francis Smart, Jean Patrick Alfred, Mamoutou Diabate, Martina Baye, Mohamed Lamine Yansane, Naod Wendrad, Nur Ali Mohamud, Paul Mbaka, Sylvain Yuma, Youssoupha Ndiaye, Husnia Sadat, Helal Uddin, Helen Kiarie, Raharison Tsihory, George Mwinnyaa, Jean de Dieu Rusatira, Pablo Amor Fernandez, Pierre Muhoza, Prativa Baral, Salomé Drouard, Tawab Hashemi, Jed Friedman, Gil Shapira

**Affiliations:** 1 The Global Financing Facility for Women, Children, and Adolescents, Washington, DC, United States of America; 2 Bloomberg School of Public Health, Johns Hopkins University, Baltimore, United States of America; 3 Development Research, The World Bank, Washington, United States of America; 4 Ghana Health Service, Accra, Ghana; 5 Ministry of Health, Lilongwe, Malawi; 6 Federal Ministry of Health, Abuja, Nigeria; 7 Ministry of Health, Monrovia, Liberia; 8 Ministry of Health and Sanitation, Freetown, Sierra Leone; 9 Ministère de la Sante publique et de la population, Port-au-Prince, Haiti; 10 Ministère de la Santé et de l’Hygiène Publique, Bamako, Mali; 11 Ministére de la Sante Publiqué, Yaoundé, Cameroon; 12 Ministère de la Sante, Conakry, Guinea; 13 Ministry of Health, Addis-Ababa, Ethiopia; 14 Federal Ministry of Health & Human Services, Mogadishu, Somalia; 15 Ministry of Health, Kampala, Uganda; 16 Ministére de la Sante, Kinshasa, Republique Democratique du Congo; 17 Ministere de la santé et de l’action sociale, Dakar, Senegal; 18 Ministry of Health and Family Welfare, Dhaka, Bangladesh; 19 Ministry of Health, Nairobi, Kenya; 20 Ministère de la Sante publique, Antananarivo, Madagascar; London School of Hygiene and Tropical Medicine, UNITED KINGDOM

## Abstract

**Background:**

The Coronavirus Disease 2019 (COVID-19) pandemic has had wide-reaching direct and indirect impacts on population health. In low- and middle-income countries, these impacts can halt progress toward reducing maternal and child mortality. This study estimates changes in health services utilization during the pandemic and the associated consequences for maternal, neonatal, and child mortality.

**Methods and findings:**

Data on service utilization from January 2018 to June 2021 were extracted from health management information systems of 18 low- and lower-middle-income countries (Afghanistan, Bangladesh, Cameroon, Democratic Republic of the Congo (DRC), Ethiopia, Ghana, Guinea, Haiti, Kenya, Liberia, Madagascar, Malawi, Mali, Nigeria, Senegal, Sierra Leone, Somalia, and Uganda). An interrupted time-series design was used to estimate the percent change in the volumes of outpatient consultations and maternal and child health services delivered during the pandemic compared to projected volumes based on prepandemic trends. The Lives Saved Tool mathematical model was used to project the impact of the service utilization disruptions on child and maternal mortality. In addition, the estimated monthly disruptions were also correlated to the monthly number of COVID-19 deaths officially reported, time since the start of the pandemic, and relative severity of mobility restrictions. Across the 18 countries, we estimate an average decline in OPD volume of 13.1% and average declines of 2.6% to 4.6% for maternal and child services. We projected that decreases in essential health service utilization between March 2020 and June 2021 were associated with 113,962 excess deaths (110,686 children under 5, and 3,276 mothers), representing 3.6% and 1.5% increases in child and maternal mortality, respectively. This excess mortality is associated with the decline in utilization of the essential health services included in the analysis, but the utilization shortfalls vary substantially between countries, health services, and over time. The largest disruptions, associated with 27.5% of the excess deaths, occurred during the second quarter of 2020, regardless of whether countries reported the highest rate of COVID-19-related mortality during the same months. There is a significant relationship between the magnitude of service disruptions and the stringency of mobility restrictions. The study is limited by the extent to which administrative data, which varies in quality across countries, can accurately capture the changes in service coverage in the population.

**Conclusions:**

Declines in healthcare utilization during the COVID-19 pandemic amplified the pandemic’s harmful impacts on health outcomes and threaten to reverse gains in reducing maternal and child mortality. As efforts and resource allocation toward prevention and treatment of COVID-19 continue, essential health services must be maintained, particularly in low- and middle-income countries.

## Abstract 2

Tashrik Ahmed and co-workers study health-care use and maternal and child health outcomes across low- and lower-middle-income countries during the COVID-19 pandemic.

## Author summary

**Why was this study done?:**

- Disruptions to essential health services during the SARS-CoV-2 (COVID-19) pandemic amplify the pandemic’s impact on morbidity and mortality and pose a profound threat to the ability of low- and middle-income countries to achieve Universal Health Coverage (UHC) and the Sustainable Development Goals (SDGs).
- While early studies projected mortality based on hypothesized scenarios, this study presents indirect child, neonatal, and maternal mortality projections during the COVID-19 pandemic based on actual service utilization data.
- This study adds to multicountry evidence on changes in the utilization of essential health services from low- and middle-income countries.

**What did the authors do and find?:**

- We used data on service utilization in 18 countries to estimate the percent change in health services delivered between March 2020 and June 2021, compared to expected levels given prepandemic trends.
- The analysis indicates that all countries experienced service disruptions, the largest of which occurred at the pandemic’s start and during months with strict mobility restrictions, regardless of the reported monthly COVID-19 mortality rates, demonstrating variation in the magnitude of disruptions across countries, services, and time.
- Using a mathematical model, we project these disruptions are associated with an additional 113,962 deaths among women in children in the 18 countries, representing increases of 3.6% in child mortality and 1.5% in maternal mortality.

**What do these findings mean?:**

- The projected excess mortality caused by disruptions to essential health services is smaller than many initial projections, though still comprises a substantial increase in mortality.
- There is strong evidence to recommend that context-specific measures to safeguard essential services be integrated into pandemic preparedness and response activities.

## Introduction

By June 2021, more than a year after the World Health Organization (WHO) declared the Coronavirus Disease 2019 (COVID-19) outbreak a pandemic, nearly 4,000,000 deaths had been reported [1]. However, analysis of excess mortality in various countries has estimated that total mortality is larger than the reported number of COVID-19-related deaths [2–5]. While the gap between excess mortality and officially reported COVID-19-related deaths is partly explained by underreporting, previous outbreaks have demonstrated that indirect health effects caused by reductions in the delivery of routine health services could be as important as the direct consequences [6]. The threat of this double crisis is particularly worrying low- and middle-income countries, which on average have higher mortality rates, more fragile health systems, and health outcomes that are more sensitive to income shocks, such as those unleashed by the COVID-19 pandemic [6,7]. These factors heighten the risk of short-term downturns in the utilization of preventive, promotive, and curative care to erode the hard-fought progress toward reducing global maternal and child mortality and lead to a prolonged secondary health crisis.

Pandemics can affect health service utilization through numerous pathways. Health systems may have reduced capacity to supply services and implement rapid adaptations due to limitations in infrastructure, health workforce, supply chains, and financial space. Limited resources to respond to a pandemic might necessitate reallocation away from routine activities and may impact the provision of essential health services through reduced clinic hours, caps on patient intake, and changes in the types of services offered. Demand-side factors, such as mobility restrictions, shutdowns of public transportation, perceived changes in quality of services, or fear of contracting COVID-19 at health facilities, may impede service accessibility and care-seeking [8]. The economic contraction caused by the pandemic may constrain the ability to pay for health services [9].

At the start of the COVID-19 pandemic, statistical models projected maternal and child mortality increases based on hypothesized service disruption scenarios [10–12]. Multiple sources have since confirmed increases in adverse maternal outcomes but lower than expected utilization of reproductive, maternal, and child (RMNCH) services during the pandemic [13–16]. Studies have quantified decreases in total health facility attendance, complementing the qualitative reports by health workers and stakeholders [13,17,18]. In Nepal, a large cohort study of women found decreases of more than half in institutional delivery rates, poorer quality of care, and increases in stillbirth and neonatal mortality rates [19]. Phone surveys of households in 39 low- and middle-income countries in April to August 2020 found that a substantial proportion of households reported forgone care [20]. While there is clear evidence that service disruptions have occurred, there is substantial variation across countries, levels of care, and service types. For example, studies from Burkina Faso, Kenya, and Mozambique found limited disruptions in contraceptive use and quick recovery to expected levels. In Bangladesh, 40% of mothers reported disruptions to family planning services [21–23]. The findings on disruptions to child vaccination programs are more consistent across countries, as many countries temporarily paused mass vaccination campaigns between March and May 2020 [24–26].

This study estimates the reductions in essential health service utilization across low- and middle-income countries and projects indirect mortality caused by the pandemic. Most studies have involved either fully hypothetical scenarios or empirical data from only a small set of health facilities for a short duration of time. We present broader evidence of the impact of the COVID-19 pandemic on health service delivery by analyzing comprehensive data from 18 countries on essential services between March 2020 and June 2021. Based on the estimated decline in service coverage, the underlying burden of disease, and the effectiveness of different interventions in preventing deaths, we aim to generate more accurate forecasts of indirect maternal and child mortality.

## Methods

We used an interrupted time-series design to estimate the percent change in the volumes of essential health services delivered during the pandemic. These estimates of lost services were translated into relative changes in coverage of interventions delivered during those periods to project the number of lives lost. The estimated monthly disruptions were also correlated to officially reported COVID-19 mortality rates, time since the start of the pandemic, and relative severity of mobility restrictions to determine which drivers are associated with changes in measured disruptions over time. The analysis was modeled on a previous study described elsewhere [27]. No changes to the analysis plan were made due to comments from reviewers or observations in the data. Data sharing agreements were established with all governments. Analysis of these secondary data did not constitute human subjects research and was considered public health practice. Thus, institutional research board approval was not required nor sought.

### Data sources and preparation

Monthly administrative data on the volume of key essential health services between January 2018 and June 2021 were collated from 18 countries participating in a monitoring activity supported by the Global Financing Facility for Women, Children, and Adolescents (GFF). Eleven countries are classified as low-income by the World Bank: Afghanistan, the Democratic Republic of Congo (DRC), Ethiopia, Guinea, Liberia, Madagascar, Malawi, Mali, Sierra Leone, Somalia, and Uganda. The other 7 countries, Bangladesh, Cameroon, Ghana, Haiti, Kenya, Nigeria, and Senegal, are classified as lower-middle-income. Seven services were selected to represent the continuum of reproductive, maternal, and child health: family planning, antenatal care initiation (ANC1), antenatal care completion (ANC4), delivery, postnatal care initiation (PNC1), bacillus Calmette–Guérin (BCG) vaccine administration, and completion of pentavalent schedule (Penta3). These services were selected because they are high completeness across countries and serve as proxies for other services and interventions delivered at the same point. In addition, outpatient consultations (OPDs) were used as a proxy for the general use of health services. Outcome measures were not included since rare outcomes (e.g., maternal death, stillbirths) are difficult to accurately capture in facility data, or the data completeness was too poor for this analysis. Seven countries’ administrative data systems were missing 1 indicator, and Uganda was missing 2 indicators. Family planning volume was the most frequently missing indicator and was not reported in 5 countries (see Table A in S1 Text). The analysis used available-case analysis, where facilities with partial facility-month observations were included in the analysis. Differences in indicator definitions were observed across countries, particularly in OPD (total attendance versus total outpatient consultations), delivery (institutional deliveries versus institutional deliveries with a skilled birth attendant), and PNC1 (first postnatal visit versus time-bound PNC visits). In countries with both versions of indicators, a sensitivity check was conducted to demonstrate that both reporting methods yielded similar results (see Tables B, C, and D in S1 Text).

HMIS data validity is often assessed in the context of measuring service coverage levels and can reflect challenges due to factors such as poor representativeness and the accuracy of population denominators [28]. Despite finding shortcomings in measuring service coverage, previous authors have called for the greater use of HMIS data, specifically the absolute number of services provided each month, in research and policy decisions. In this study, we do not attempt to estimate population service coverage but rather assume that the change in service-specific utilization reported by facilities in the HMIS represents the percentage change in population service coverage. We believe this use of HMIS, not as an estimate of coverage but as an estimate of coverage change, is less subject to various potential biases. The ability to rigorously estimate changes in service volume despite limitations of facility data has been previously demonstrated [29]. With the exception of Bangladesh and Nigeria, there should be high representativeness of facilities that report to HMIS since the public sector delivers the majority of care. The primary concern is the possible differential change in utilization between reporting and nonreporting facilities. Findings from household surveys and interviews with key health system stakeholders during the pandemic confirm that private facilities and community programs did not compensate for the disruptions in the public sector, and there were substantial levels of foregone care in the population [17,20].

HMIS data were downloaded on 22 August 2021, and were prepared for analysis by removing outlier values and restricting data for indicators with low completeness. These preparation steps are detailed in the Supporting information (see Text A in S1 Text), and the advantages and disadvantages of HMIS data are discussed in previous work [14]. To further assess the quality and reliability of the data, we present a range of sensitivity tests; we describe data reporting completeness (see Fig A in S1 Text) and include a sensitivity check showing that changes in reporting patterns did not drive the results. We also specify for each country and indicator the dates dropped due to poor completeness, or data availability that may reduce the prepandemic follow-up (see Table L in S1 Text). The final dataset included 137,192 health facilities ranging from 478 facilities in Guinea to 34,701 in Nigeria. The reports cover 42 months and 8 services, for 21,421,125 nonmissing facility-month-service observations in the 18 countries.

We obtained data from 2 additional sources to assess whether service disruptions correlate with officially reported COVID-related death rates or with mobility restrictions. Data on reported COVID-19 deaths were obtained from the Center for Systems Science and Engineering (CSSE) at Johns Hopkins University, which compiles data from official government COVID-19 surveillance reports. Official accounts are likely to underreport actual mortality in settings with limited testing capacity, particularly at the beginning of the pandemic [30]. However, even if the official reports are inaccurate, there are many mechanisms through which these reports may affect service utilization, such as changing perceptions by health providers and the population on the state of the outbreak. Therefore, one way to interpret these data is as a proxy for the perceived risk of infection. Information on the policy measures affecting population mobility was obtained from the Oxford COVID-19 Government Policy Tracker, which systematically tracks implementation dates and scores the stringency of policy interventions. We selected a subset of policies that may affect population access to health facilities: public transport closures, stay-at-home requirements, movement limitations, school closures, and workplace closures. The dataset includes ordinal severity scores for each policy to capture the stringency of restriction, ranging from no restrictions to recommendations to requirements with minimal exceptions. We constructed an index representing the daily severity of mobility restrictions using the first component of a principal component analysis of these selected indicators. There is a correlation of 0.92 between the index we construct and the Oxford response stringency index, composed of a wider set of interventions. The Oxford COVID-19 Policy Tracker captures as-written strictness of policies but not levels of enforcement. As we are unaware of a reliable source on levels of enforcement of restrictions, differences in levels of enforcement between countries were not taken into account.

### Analysis of service utilization disruptions

We used an interrupted time-series approach to predict the volume of services that would have been delivered had the pandemic not occurred. The interruption period starts with the WHO pandemic declaration in March 2020, coinciding with the start of community transmission and mobility restrictions in most countries. Service and countries were modeled separately using a linear regression equation with the following form:

$$Y_{tf}=\beta_{0}+\beta_{1}T+\beta_{2..12}Month+\beta_{13..29}{PandemicMonth}_{t}+\alpha_{f}+\varepsilon_{tf}$$

where *Y*_*tf*_ is the service volume reported by facility *f* in month *t*. *T* represents the time in months since January 2018 to account for a linear secular trend (*β*_1_), *Month* represents calendar months to account for seasonality (*β*_2..12_), and *α*_*f*_ represents the facility-level fixed effect accounting for time-invariant facility characteristics. Fixed effects were replaced with facility characteristic covariates (province and facility type) in Uganda, where an update to the administrative system did not allow for consistent identification of facilities over time. *PandemicMonth* denotes a series of dummy variables for each of the months between March 2020 and June 2021. That is, *β*_13..29_ contain estimated disruption for each month since the pandemic.

To calculate the percentage change in service utilization during the pandemic months, we first used the estimation results to calculate the expected volume in the absence of the pandemic (*counterfactual*). Then, we divided the reported volumes by these expectations. The cumulative shortfall was estimated using the same model with a single pandemic period. A 2-year prepandemic time horizon was chosen to minimize confounding from changes in data collection practices, policy changes, or other health shocks while still allowing separation of seasonality effects from secular trends.

### Correlate drivers of utilization changes

We further examined whether the estimated monthly changes in service volumes showed statistical association with the time elapsed since the start of the pandemic, the monthly number of official reported COVID deaths, and the stringency of mobility restrictions. These relationships were assessed by running the following linear regression separately for each service:

$$D_{tc}=\gamma_{0}+\gamma_{1}Q+\gamma_{2}{CovidMortality}_{tc}+\gamma_{3}{RestrictionStringency}_{tc}+\alpha_{c}+\varepsilon_{tc},$$

where *D*_*tc*_ is the estimated monthly change in service volume for month *t* in country *c*. *CovidMortality*_*tc*_ represents the officially reported monthly number of COVID-19-related deaths per 100,000 people, and *RestrictionStringency*_*tc*_ represents the average monthly stringency of the mobility restrictions. *α*_*c*_ represents a country fixed effect, and *ε*_*tc*_ is a normally distributed error term.

### Mortality estimates

We estimated the impact of the service utilization disruptions on the absolute number of child, neonatal, and maternal deaths using the Lives Saved Tool (LiST). LiST is a mathematical model that forecasts mortality estimates from the coverage of 70+ RMNCH+N health interventions, considering the specific demographic and epidemiological context of a country [31]. We assumed that the relative change in the coverage of the interventions included in the LiST model was the same as the estimated relative changes in service utilization. Each intervention was linked to the service during which the intervention is typically delivered or proxied by the service assumed to have a similar utilization pattern. For interventions without a reasonable proxy, such as child nutrition services, the conservative default assumed no change in the intervention coverage. This linking of service indicators to LiST interventions is described in Table E of S1 Text. As multiple RMNCH interventions were linked to a small set of indicators, small variations in the few service indicators significantly affect the overall mortality results. To address this, we ran a sensitivity analysis using different linking combinations to understand how these linking decisions alter the results and limit their potential effect.

For each LiST intervention and country, we obtained coverage values from the most recent household survey for the country (typically a DHS or MICS), which we took as the coverage value that we would have expected in the absence of the pandemic (i.e., as a “counterfactual”). To estimate the coverage value during the pandemic, we multiplied the counterfactual coverage value by the estimated disruption of the service (proxy) indicator. This approach assumes that, during the pandemic, the change in population-level coverage was proportional to the change in reported facility-level utilization. In this way, we obtained an estimated coverage value for each intervention, country, and period. We used 3-month periods (quarters), aggregating the service disruption for the relevant proxy indicator for each quarter and calculating disrupted coverage values for each quarter of the pandemic for each country and intervention.

We ran 2 LiST analyses for each country and quarter: first, a “without pandemic” scenario, using only the counterfactual coverage values, to obtain the expected deaths in the absence of the pandemic; and second, a “with pandemic” scenario, to obtain the expected deaths during the pandemic. LiST only takes yearly input values, so we entered quarterly values as yearly values (for 2020 or 2021, as appropriate), and divided the resulting expected deaths by 4, to obtain the expected deaths for the quarter. We took the difference in expected deaths between the “with pandemic” and “without pandemic” scenarios to represent the additional deaths due to the pandemic. For each country and age group, we report the number of deaths that we would have expected in the absence of the pandemic for the period March 2020 to June 2021, the estimated number of additional maternal and child (including neonatal) deaths due to the change in service utilization during this same period, and the relative increase in mortality because of service utilization declines during the pandemic. The RECORD checklist is included in the Supporting information (S1 RECORD Checklist). Study limitations include the assumption that the changes in service-specific utilization reported in the HMIS represents the percentage of service coverage change in the population, and the inability to account for differential changes in health-seeking behaviors across severity of need.

## Results

A total of 137,192 facilities were included in the analysis, as shown in Table 1, though the number of facilities reporting information for various health services varied by country. The following sections describe findings of service disruptions, regression analysis, and LiST modeling, respectively.

**Table 1:** Sample size used in primary analysis (number of facilities).

	Facility Level	Afghanistan	Bangladesh	Cameroon	DRC	Ethiopia	Ghana	Guinea	Haiti	Kenya	Liberia	Madagascar	Malawi	Mali	Nigeria	Senegal	Sierra Leone	Somalia	Uganda*	Grand Total
ANC1	Hospital	125	109	317	371	435	494	17	62	649	31	3	79		2,916	17	18	51	176	5,870
	Health Center	1,505	649	5,164	11,429	4,285	1,858	315	310	1,369	55	2,747	513		13,974	124	258	531	1,815	46,901
	Lower level	863		131	5,200	12,526	2,887	103	356	4,829	510		83		12,585	1,546	1,097	61	1,711	44,488
	Other/Unknown		55	325	1,088		73	2	69	237	7	484	8			107		17	198	2,670
ANC4	Hospital	119	104	314	364	435	491	16	62	771	30	3	79	2	2,673	17	14	50	169	5,713
	Health Center	1,489	632	4,933	11,255	4,294	1,834	316	285	1,662	54	2,769	513	1,529	13,163	124	258	523	1,648	47,281
	Lower level	846		124	4,887	13,028	2,770	103	290	5,957	494		81	247	11,256	1,546	1,096	58	1,673	44,456
	Other/Unknown		48	315	1,046		71	2	60	453	6	484	8	9		106		16	151	2,775
BCG	Hospital	132			91		329	15	59		31	3	74		2,216	2	18	56	174	3,200
	Health Center	1,152	601		10,642		1,323	315	286		54	2,756	532	1,504	13,859	113	257	486	2,031	35,911
	Lower level	851	56		2,503		5,286	102	335		487		92	6	13,619	1,472	1,091	56	1,644	27,600
	Other/Unknown				213		80	1	61		7	221	3			48		17	89	740
DELIVERY	Hospital	128	104	313	444	406	491	14	59	754	31	1	77	2	2,750	5	25	52	171	5,827
	Health Center	1,488	656	5,045	11,437	3,709	1,765	305	181	1,462	53	2,692	470	1,507	12,278	114	249	495	1,043	44,949
	Lower level	828	18	131	5,764	3	1,973	99	164	4,094	495		36	228	9,092	1,487	1,087	35	1,632	27,166
	Other/Unknown		58	311	1,211		64	2	39	251	7	353	6	8		61		14	146	2,531
FP	Hospital	128	2	280		427	293	41	58	752	29	2	64	1	2,226			34	83	4,420
	Health Center	1,501	69	4,472		4,344	1,439	304	295	1,763	48	2,767	517	1,492	13,050			121	1,357	33,539
	Lower level	862	12,250	92		15,287	5,012	99	336	6,742	484		118	193	11,706			6	1,010	54,197
	Other/Unknown			245			75	1	66	575	8	496	17	8				2	146	1,639
OPD	Hospital	145	125	322	471	439	516	37	70	822	34	4	75	1	3,159	18	44	52	189	6,523
	Health Center	1,509	499	5,317	11,707	4,393	2,163	17	323	1,891	58	2,807	537	1,537	14,671	128	253	534	2,946	51,290
	Lower level	872	13,699	133	6,626	15,591	3,027		385	7,299	556		100	269	15,993	1,577	1,096	64	1,795	69,082
	Other/Unknown			361	1,317		96	2	76	725	10	876	4	11		152		17	480	4,127
PENTA3	Hospital	126		281	101	362	336	15	59	730	32	3	74		2,201	3	18	56	177	4,574
	Health Center	1,176	598	4,399	10,818	4,138	1,413	315	290	1,528	54	2,759	532	1,501	13,884	114	257	490	2,162	46,428
	Lower level	857	56	88	2,714	15,619	6,311	102	338	5,535	489		93	6	13,674	1,484	1,092	59	1,669	50,186
	Other/Unknown			255	240		86	1	61	317	7	256	4			54		18	104	1,403
PNC1	Hospital	125	105	311	436	435	492	3	55	742	30	1	58	2	2,356	7	21	48		5,227
	Health Center	1,505	630	4,809	11,396	4,334	1,780	13	246	1,645	52	2,581	346	1,518	11,943	121	253	523		43,695
	Lower level	863	12,728	120	5,653	14,467	2,408		284	5,932	469		27	220	9,548	1,521	1,082	57		55,379
	Other/Unknown		58	293	1,185		66		49	439	5	228	1	8		71		17		2,420
	Total	2,528	14,847	6,214	20,150	20,739	9,323	478	861	10,899	659	3,705	783	1,847	34,701	1,920	1,406	680	5,452	137,192

*Uganda sample size is given for January 2021, as facilities do not have consistent ID codes (see Table L of S1 Text).

DRC is Democratic Republic of the Congo. ANC1 refers to the first antenatal care visit. ANC4 refers to the fourth antenatal care visit. BCG refers to bacillus Calmette–Guérin vaccination. FP refers to family planning consultations. OPD refers to outpatient visits. Penta3 refers to the third dose of pentavalent vaccine. PNC1 refers to the first postnatal care visit.

### Service disruptions

**Fig 1:**
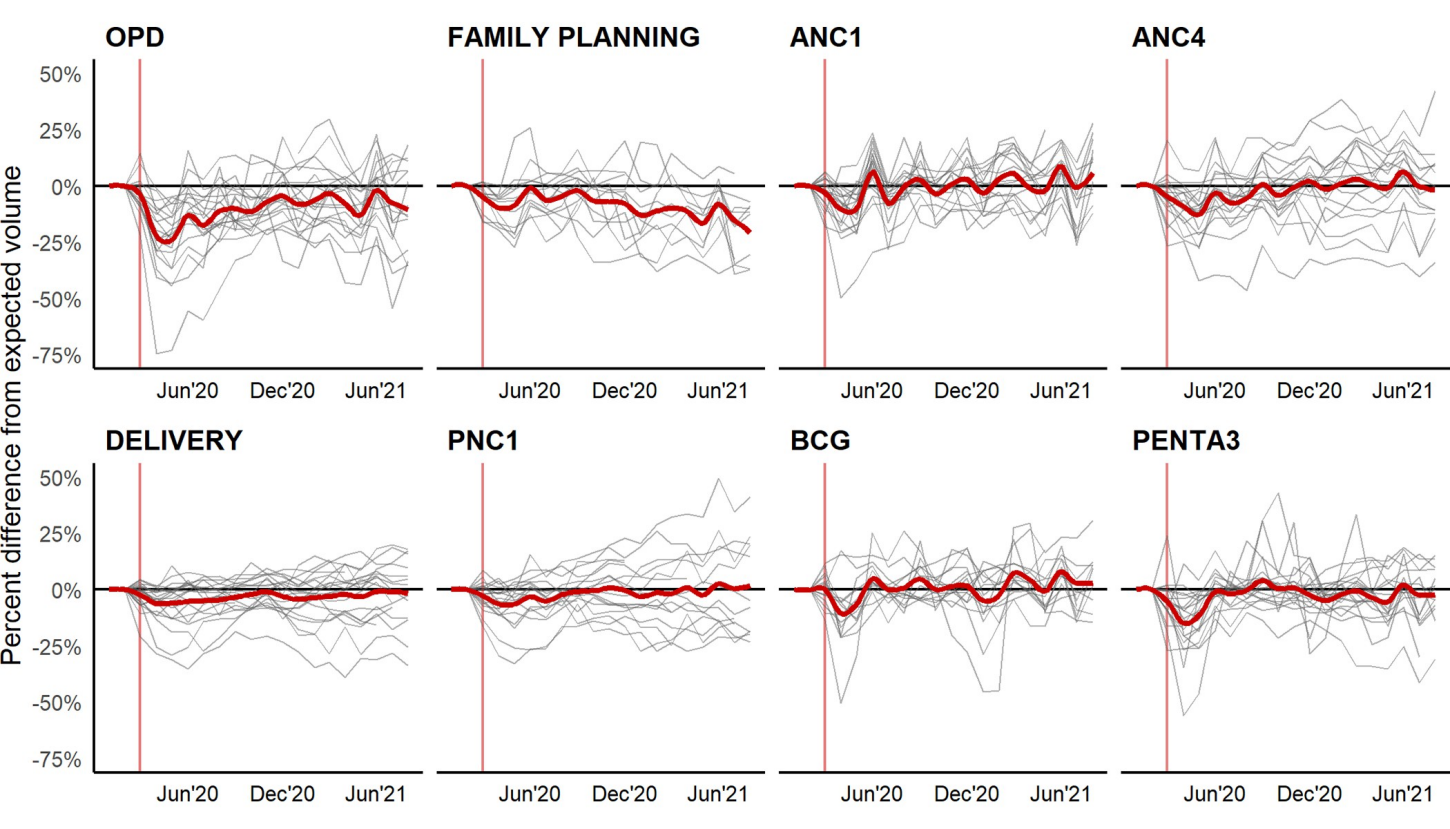
Percent change in volume from expected levels based on prepandemic trends by selected health services across 18 countries, March 2020–February 2021. Note: The horizontal line at 0% represents the expected volume of services based on prepandemic trends. The gray lines plot country-specific changes in service utilization. The monthly country-specific results are presented in Table A in S1 Text. The red line is a multicountry unweighted moving average of the change in utilization plotted by a locally estimated scatterplot smoothing (LOESS) regression. Details on indicator reporting for each country can be found in Table L in S1 Text. ANC1 refers to First Antenatal Care Visit. ANC4 refers to the Fourth Antenatal Care Visit. BCG refers to bacillus Calmette–Guérin vaccination. OPD refers to Outpatient visits. Penta3 refers to the Third dose of Pentavalent vaccine. PNC1 refers to First Postnatal Care Visit.

We focus first on the number of outpatient consultations as a proxy for general service utilization. As shown in Table 2, the cumulative number of reported outpatient consultations between March 2020 and June 2021 is significantly lower than expected, given the prepandemic trends in all countries apart from Cameroon, Liberia, and Somalia. These 3 countries also experienced substantial monthly declines in outpatient consultations compared to expected values, but the reductions over the 16-month duration are not statistically significant (see Table A in S1 Text). On average, the countries in this analysis experienced a cumulative reduction of 13% in outpatient consultations compared to historical utilization trends. The largest decline of 40% is estimated for Bangladesh, followed by 25% in Haiti and Kenya. Large declines between 10% and 20% are estimated for Ethiopia, Ghana, Guinea, Madagascar, Nigeria, Senegal, Sierra Leone, and Uganda. As seen in Fig 1, unweighted moving average monthly outpatient service volumes are below expected for all months between March 2020 and June 2021.

**Table 2:** Cumulative change in service volumes during the pandemic period (March 2020–June 2021) compared to expected volume based on prepandemic trends.

Country	Outpatient consultation (OPD)	Family Planning (FP)	First Antenatal Care Visit (ANC1)	Fourth Antenatal Care Visit (ANC4)	Delivery	First Postnatal Care Visit (PNC1)	BCG Vaccination	Third Dose of the Pentavalent Vaccination (Penta3)
Afghanistan	−9.0%***	−6.3%*	−8.0%***	−6.1%*	−9.4%**	−8.0%**	3.00%	−3.30%
	(−12.5%, −5.4%)	(−13.6%, 1.1%)	(−12.1%, −3.8%)	(−12.9%, 0.7%)	(−15.6%, −3.2%)	(−13.3%, −2.8%)	(−1.7%, 7.8%)	(−10.6%, 4%)
Bangladesh	−40.0%***	-	−26.2%***	−33.1%***	−11.40%	−19.6%***	−3.5%*	−12.9%***
	(−47%, −32.9%)		(−34.2%, −18.2%)	(−46.7%, −19.5%)	(−28.9%, 6%)	(−27.4%, −11.7%)	(−7.1%, 0.1%)	(−16.8%, −9.1%)
Cameroon	−2.70%	-	1.50%	−2.30%	2.3%**	4.0%**	-	−0.40%
	(−6%, 0.7%)		(−0.6%, 3.6%)	(−6.7%, 2.1%)	(0.1%, 4.5%)	(0.7%, 7.3%)		(−3.3%, 2.4%)
DRC	−4.4%***	-	1.3%**	2.0%**	−1.0%*	−1.2%**	-	−0.10%
	(−6.6%, −2.1%)		(0.4%, 2.2%)	(0.7%, 3.2%)	(−2%, 0.1%)	(−2.3%, −0.2%)		(−1.1%, 0.9%)
Ethiopia	−15.4%***	1.10%	−4.2%***	−3.4%***	2.0%**	1.10%	-	−2.6%***
	(−18.6%, −12.3%)	(−0.8%, 3%)	(−5.5%, −2.8%)	(−5.2%, −1.5%)	(0.3%, 3.7%)	(−0.8%, 2.9%)		(−4%, −1.2%)
Ghana	−18.1%***	-	4.7%***	−0.20%	−0.30%	1.40%	−3.7%***	−0.70%
	(−20.8%, −15.3%)		(3.5%, 5.8%)	(−3.3%, 2.9%)	(−1.9%, 1.3%)	(−0.8%, 3.7%)	(−5.5%, −1.9%)	(−3.3%, 1.8%)
Guinea	−14.5%**	−10.4%**	−0.20%	−6.1%**	0.50%	-	−4.2%**	−5.8%**
	(−24.8%, −4.2%)	(−18.5%, −2.3%)	(−3.8%, 3.4%)	(−10.7%, −1.5%)	(−4%, 5.1%)		(−7.5%, −0.9%)	(−10.2%, −1.5%)
Haiti	−25.0%***	0.30%	−7.5%**	−14.4%**	−25.5%***	−19.0%***	−12.0%***	3.80%
	(−32.3%, −17.8%)	(−7.7%, 8.2%)	(−13.1%, −1.8%)	(−22.7%, −6.1%)	(−34%, −17.1%)	(−26%, −11.9%)	(−17.2%, −6.7%)	(−6.6%, 14.2%)
Kenya	−24.9%***	0.20%	−3.9%***	−13.5%***	−4.2%**	−4.5%**	-	−0.30%
	(−27.2%, −22.5%)	(−2.1%, 2.5%)	(−5.6%, −2.1%)	(−16.9%, −10.2%)	(−7%, −1.3%)	(−7.8%, −1.1%)		(−1.8%, 1.2%)
Liberia	−6.00%	−2.80%	−4.0%*	−7.90%	−3.8%**	−7.5%***	2.60%	−2.60%
	(−14.5%, 2.5%)	(−12.8%, 7.1%)	(−8.7%, 0.7%)	(−17.9%, 2%)	(−6.9%, −0.7%)	(−11.6%, −3.4%)	(−1.3%, 6.5%)	(−7.9%, 2.7%)
Madagascar	−10.0%***	−2.30%	0.40%	3.80%	5.5%**	8.7%***	0.50%	−2.6%*
	(−15.2%, −4.9%)	(−8.4%, 3.8%)	(−1.9%, 2.8%)	(−0.8%, 8.3%)	(2.2%, 8.8%)	(3.9%, 13.6%)	(−3.3%, 4.3%)	(−5.6%, 0.4%)
Malawi	−7.0%**	4.40%	−2.00%	3.1%*	−3.6%**	−2.80%	−1.30%	−3.5%**
	(−13.2%, −0.9%)	(−3.3%, 12.2%)	(−6.7%, 2.6%)	(−0.5%, 6.7%)	(−6.7%, −0.5%)	(−8.4%, 2.8%)	(−4.9%, 2.2%)	(−6%, −1%)
Mali	−5.0%**	−9.8%**	-	-	−3.4%*	−0.50%	−10.0%***	−12.5%***
	(−8.8%, −1.3%)	(−19.1%, −0.5%)			(−7.3%, 0.5%)	(−3.2%, 2.1%)	(−13.5%, −6.4%)	(−16.6%, −8.4%)
Nigeria	−14.3%***	−4.6%***	−5.4%***	−14.5%***	−7.9%***	−1.80%	1.00%	−2.3%**
	(−16.8%, −11.7%)	(−6.9%, −2.2%)	(−7.5%, −3.3%)	(−18.9%, −10.2%)	(−11.1%, −4.7%)	(−7.5%, 3.9%)	(−0.6%, 2.7%)	(−3.9%, −0.8%)
Senegal	−13.3%***	-	−12.3%***	1.00%	4.2%**	3.70%	4.3%**	−8.8%***
	(−19.2%, −7.4%)		(−15.1%, −9.5%)	(−1.2%, 3.1%)	(1%, 7.3%)	(−0.9%, 8.2%)	(1.1%, 7.4%)	(−11.2%, −6.3%)
Sierra Leone	−13.6%***	−19.2%***	−1.60%	5.3%**	−4.7%**	−4.4%**	−6.8%**	−9.5%**
	(−17.3%, −9.9%)	(−24.2%, −14.2%)	(−4.8%, 1.7%)	(0.4%, 10.2%)	(−7.6%, −1.9%)	(−7.5%, −1.3%)	(−10.6%, −3%)	(−14.7%, −4.3%)
Somalia	4.00%	-	1.10%	11.4%**	1.10%	8.6%**	−3.70%	−3.60%
	(−1.8%, 9.8%)		(−3.6%, 5.8%)	(3.5%, 19.2%)	(−3.9%, 6%)	(8.6%, 1.1%)	(−9.1%, 1.6%)	(−9.3%, 2%)
Uganda	−17.2%***	-	−2.8%**	−4.2%**	−6.3%***	-	−7.7%***	−6.3%***
	(−20%, −14.4%)		(−5.6%, 0%)	(−7.4%, −1%)	(−8.9%, −3.7%)		(−10.6%, −4.8%)	(−8.9%, −3.6%)
**Mean**	−13.1%	−4.4%	−4.1%	−4.6%	−3.7%	−2.6%	−3.0%	−4.1%

The table presents regression coefficients and the 95% confidence intervals in parentheses. Changes in service volumes are estimated using an interrupted time-series approach. The reported mean is the average value across countries and is not population weighted. Details on indicator reporting can be found in Table L in S1 Text. Sample sizes by country and indicator are reported in Table 1.

*P* values are calculated using *t* tests, with the magnitude indicated by asterisks as follows

*p < 0.1

** *p* < 0.05

*** *p* < 0.001.

The disruptions to RMNCH services are smaller on average than those observed in outpatient consultations. For child vaccination, 10 out of 18 countries experienced significant cumulative reductions in the number of children receiving the third dose of the pentavalent vaccine. Out of the 14 countries with HMIS data on administered BCG vaccine doses, 8 experienced significant cumulative reductions. In most countries, the monthly reductions in vaccination were largest at the start of the pandemic and returned to the prepandemic expectation by July 2020 (see Fig 1). While the return to the expected levels is encouraging, we do not see an increase representing facility-based catch-up for the vaccinations missed early during the pandemic. A different pattern is observed for antenatal care initiation (ANC1). An initial decrease is followed by an increase above the expected volume, indicating that some women may have delayed their visit without completely forgoing antenatal care. Reproductive and maternal health services disruptions were more context-specific than disruptions in outpatient care and child vaccination. Significant declines in the delivery indicators, for example, are estimated in 10 out of the 18 countries. At the same time, Cameroon, Ethiopia, Madagascar, and Senegal reported volumes significantly exceeding those expected based on prepandemic trends. Significant cumulative reductions in family planning services are estimated for 6 out of 12 countries with available data. Large reductions in family planning volume of at least 10% were experienced in Guinea, Mali, and Sierra Leone.

### Correlates

**Fig 2:**
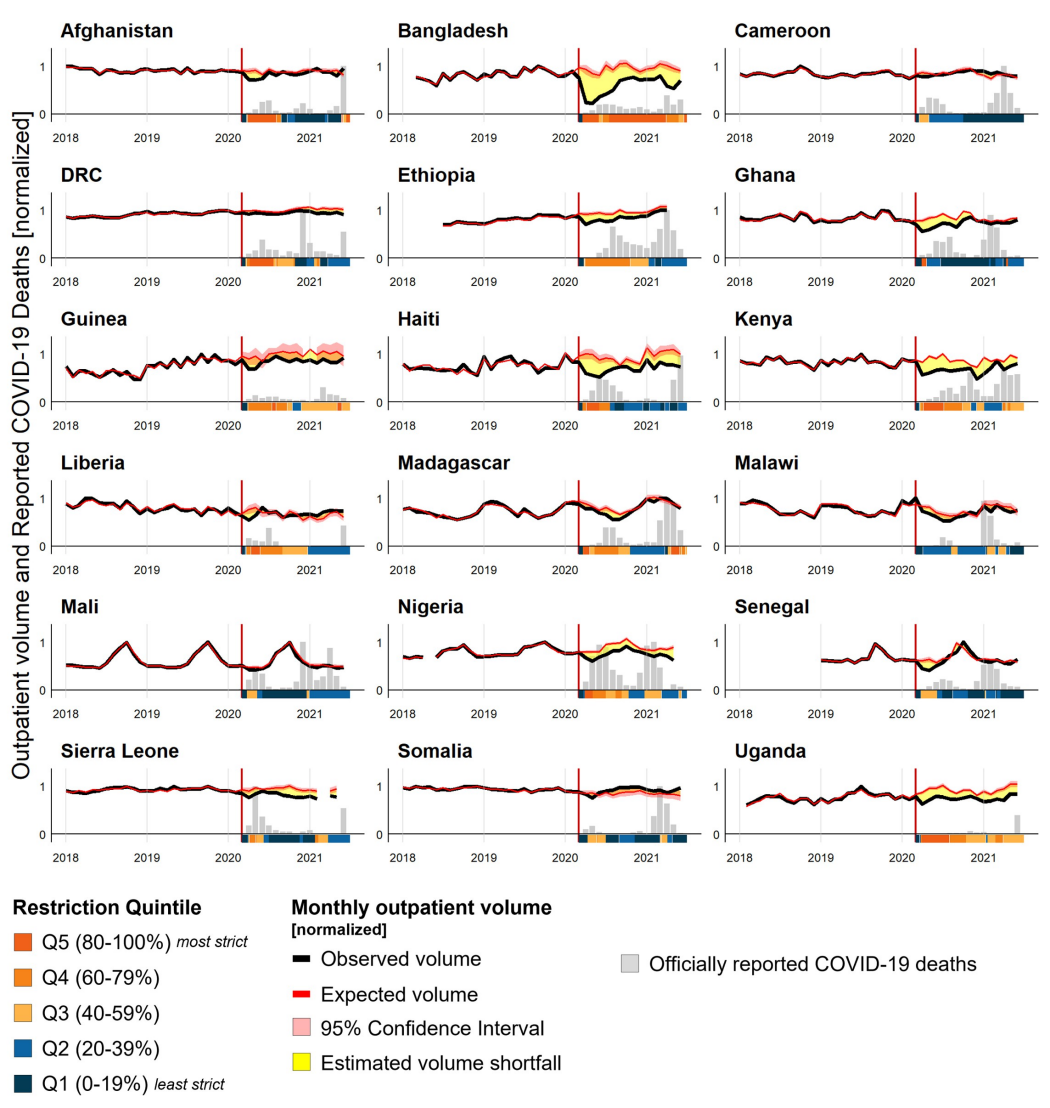
Estimated and observed volume of outpatient consultations with officially reported COVID-19 deaths per 100,000 and mobility restrictions by country, January 2018–June 2021. **Note:** Outpatient consultations are used as a proxy for the utilization of general health services. Data on officially reported COVID-19 deaths are compiled from Johns Hopkins University Coronavirus dashboard [1]. Population denominators for all countries are based on 2019 estimates from the World Bank Development Indicators database. Utilization volume and mortality data are normalized across countries by dividing by the highest observed monthly value within each country. Data on mobility restrictions is summarized by an index of public transport closures, stay-at-home requirements, movement limitations, school closures, and workplace closures stringency scores provided by the Oxford COVID-19 Government Response Tracker. The scores from this index are normalized, and the categorized into quintiles. Gaps in the service volume data are due to months removed because of low completeness. Details on indicator reporting can be found in Table L in S1 Text and data completeness can be found in Fig A in S1 Text. DRC is Democratic Republic of the Congo. Results for ANC1, delivery, BCG, and Penta3 are visualized in Fig B in S1 Text.

In addition to the cross-country variation, the magnitude of service volume disruption varied during the pandemic. Fig 2 presents the example of outpatient consultations and portrays a country-specific relationship between the magnitude of the estimated disruptions, the time elapsed since the beginning of the pandemic, and the monthly number of reported COVID-related deaths. To assess the correlations of the disruptions with these factors, we present cross-country regression results in Table 3. The largest utilization reductions were experienced in April and May 2020, and this decline is unrelated to pandemic severity as proxied by reported COVID-19-related mortality. Family planning is the only service with large drops in the second quarter of 2021. Moreover, there is no significant relationship between the number of monthly officially reported COVID-19-related deaths and the magnitude of change in any service. There is, however, a significant relationship between imposed restrictions and the magnitude of the estimated disruptions in outpatient consultations, child vaccinations, and the fourth antenatal care visit. For example, a standard deviation in the mobility restrictions stringency is associated with a 3.9% reduction in outpatient consultation volume (Column 1 of Table 3).

**Table 3:** Correlates of monthly disruption magnitude in service volume.

Dependent variable: percentage monthly decline in outpatient consultations volume								
	OPD	FP	ANC1	ANC4	Delivery	PNC1	BCG	Penta3
March–May 2020	−6.10***	4.61*	−9.27***	−7.23***	−1.86*	−4.25***	−6.91***	−6.72***
	(−9.77, –2.43)	(−0.38, –9.61)	(−12.87, –5.66)	(−10.53, –3.93)	(−3.90, 0.19)	(−7.32, –1.18)	(−11.36, –2.45)	(−10.46, –2.98)
June–Aug 2020	−2.58	8.34***	−2.55	−3.968**	−1.10	−2.73*	1.05	2.71
	(−6.11, 0.96)	(3.41, 13.28)	(−6.05, 0.94)	(−7.16, –0.78)	(−3.07, 0.87)	(−5.63, 0.18)	(−3.21, 5.30)	(−0.89, 6.31)
Sep–Nov 2020	−0.91	4.42*	−2.22	−2.13	0.18	−0.55	−0.17	4.82***
	(−4.26, 2.43)	(−0.13, 8.97)	(−5.49, 1.04)	(−5.13, 0.87)	(−1.68, 2.04)	(−3.31, 2.21)	(−4.28, 3.95)	(1.41, 8.23)
Dec 2020–Feb 2021	0.21	−0.55	−1.51	−1.38	−0.86	−1.30	−5.13**	−1.63
	(−3.03, 3.45)	(−5.1, 4.01)	(−4.68, 1.67)	(−4.28, 1.52)	(−2.66, 0.95)	(−3.99, 1.38)	(−9.14, –1.12)	(−4.93, 1.68)
Officially reported deaths per 100k people^a^	0.06	0.03	−0.03	−0.10	−0.04	−0.08	0.02	0.10
	(−0.16, 0.28)	(−0.23, 0.29)	(−0.23, 0.17)	(−0.29, 0.09)	(−0.16, 0.08)	(−0.25, 0.09)	(−0.23, 0.27)	(−0.12, 0.32)
Index of mobility	−3.87***	−2.31*	−0.73	−1.98**	−0.49	−0.56	−2.27**	−2.67***
Restrictions stringency ^b^	(−5.67, –2.07)	(−4.70, 0.08)	(−2.46, 1.00)	(−3.57, –0.39)	(−1.50, 0.51)	(−2.04, 0.92)	(−4.49, –0.06)	(−4.50, –0.83)
Number of observations	279	163	257	263	279	241	217	279

The table presents regression coefficients and the 95% confidence intervals in parentheses. The dependent variable is the estimated percentage monthly change in volume of outpatient consultations for a given service, presented in Fig 1 and in Table A in S1 Text. All regressions include country fixed effects. Details on indicator reporting can be found in Table L in S1 Text. DRC is Democratic Republic of the Congo. ANC1 refers to the first antenatal care visit. ANC4 refers to the fourth antenatal care visit. BCG refers to bacillus Calmette–Guérin vaccination. FP refers to family planning consultations. OPD refers to outpatient visits. Penta3 refers to the third dose of pentavalent vaccine. PNC1 refers to the first postnatal care visit.

^a^Reported COVID-19 deaths were obtained from the CSSE at Johns Hopkins University. The monthly number of COVID-19-attributable deaths is population standardized per 100,000 people using the 2019 World Development Report estimated population.

^b^An index of mobility restrictions stringency is constructed with principal component analysis using data from the Oxford COVID-19 Government Policy Tracker on daily restriction. An average over the daily stringency is taken to compute the monthly index.

*P* values are calculated using *t* tests, with the magnitude indicated by asterisks as follows: *p < 0.1; **p < 0.05; ***p < 0.001.

### Mortality estimates

Table 4 shows estimates of the impact of service disruptions on child, neonatal, and maternal mortality. The country with the greatest estimated increase in mortality was Bangladesh, with a 14.9% increase in child mortality, 9.7% increase in neonatal mortality, and 3.9% increase in maternal mortality. Haiti, Kenya, Nigeria, Sierra Leone, and Uganda were also estimated to have child mortality increases of 5% or greater. Cameroon, Liberia, and Somalia were estimated to have small reductions in child mortality, and 6 countries were estimated to have minor reductions in maternal mortality. We estimate that 27.6% of the additional child deaths and 24.3% of the additional maternal deaths occurred due to utilization declines in Quarter 2 of 2020, reflecting the above results (see Table G in S1 Text). In sum, the absolute number of additional deaths across the 18 countries from March 2020 to June 2021 is estimated to be 110,686 child deaths (0 to 59 months), 32,061 neonatal deaths (<1 month), and 3,276 maternal deaths. Many factors, including population size and baseline mortality rate, drive the absolute number of additional deaths. In general, estimated increases in maternal mortality across all countries were smaller than increases in child or neonatal mortality due to smaller facility delivery reductions than those in outpatient and vaccination services.

**Table 4:** Expected and additional child, neonatal, and maternal deaths, March 2020 to June 2021.

	Child deaths (0–59 months)			Neonatal deaths (<1 month)			Maternal deaths		
Country	Expected deaths	Additional deaths	Relative increase in mortality	Expected deaths	Additional deaths	Relative increase in mortality	Expected deaths	Additional deaths	Relative increase in mortality
Afghanistan	100,472	3,091	3.1%	67,835	2,238	3.3%	11,667	435	3.7%
Bangladesh	118,156	17,578	14.9%	75,257	7,317	9.7%	7,605	300	3.9%
Cameroon	89,319	−609	−0.7%	36,249	−602	−1.7%	7,219	−122	−1.7%
DRC	414,800	4,928	1.2%	153,158	2,352	1.5%	25,615	0	0.0%
Ethiopia	264,992	3,267	1.2%	153,851	664	0.4%	21,858	−76	−0.3%
Ghana	56,105	1,607	2.9%	31,996	695	2.2%	4,111	−51	−1.2%
Guinea	61,910	1,728	2.8%	22,115	332	1.5%	4,085	2	0.1%
Haiti	23,296	1,396	6.0%	10,636	703	6.6%	1,962	139	7.1%
Kenya	79,842	4,328	5.4%	44,367	1,578	3.6%	7,758	57	0.7%
Liberia	15,145	−120	−0.8%	5,970	36	0.6%	1,610	20	1.2%
Madagascar	62,158	601	1.0%	27,100	−75	−0.3%	4,399	−41	−0.9%
Malawi	40,202	1,747	4.3%	21,394	1,119	5.2%	3,335	136	4.1%
Mali	103,991	1,194	1.1%	40,264	396	1.0%	6,914	0	0.0%
Nigeria	1,194,118	60,307	5.1%	409,318	11,827	2.9%	103,980	2,261	2.2%
Senegal	31,762	1,285	4.0%	17,097	1,051	6.1%	2,606	168	6.5%
Sierra Leone	36,034	2,296	6.4%	12,886	561	4.4%	4,385	32	0.7%
Somalia	106,149	−613	−0.6%	37,779	−289	−0.8%	8,328	−13	−0.2%
Uganda	101,096	6,675	6.6%	49,924	2,158	4.3%	9,411	29	0.3%
Total	2,899,546	110,686	3.6%	1,217,196	32,061	2.8%	236,851	3,276	1.5%

Expected deaths come from estimates from the UN Inter-Agency Group for Child Mortality Estimation (child and neonatal mortality) and WHO (maternal mortality). Additional deaths are projected from the LiST mathematical model, which estimates changes in mortality from changes in intervention coverage (more information and projection methods can be found at https://www.livessavedtool.org/). Child deaths are inclusive of neonatal deaths. Additional information on these results are available in the Supporting information: Table E in S1 Text summarizes the mapping between service indicators and interventions included in LiST. Table G in S1 Text provides the detailed breakdown of this table by calendar quarter. Table H in S1 Text reports these results using the 95% confidence interval generated from the service disruptions analysis in Table 2 to bound the mortality estimates. Table I in S1 Text provides sensitivity analyses on the mapping assumptions.

We conducted 2 sensitivity analyses to understand the potential error in the mortality results. First, we used the upper and lower 95% confidence intervals of the service disruption estimates and found that the additional deaths could be 43.8% higher or 42.9% lower than the estimates in Table 4. Given the perfect correlation in the error of the disruption estimates that this approach assumes, these bounds are overly conservative. Second, we varied the linkage of service indicators to LiST interventions by setting all interventions to each service in turn and randomizing the link between interventions and services. We found that the mortality estimates could be up to 31.8% higher or 55.1% lower than Table 4. However, given the extreme assumptions that we tested related to the linkage between the HMIS indicators and the LiST interventions, these bounds are also conservative. For more detail on these sensitivity analyses, see Tables G and H in S1 Text.

## Discussion

Compared to the expected volumes based on prepandemic trends, we estimate statistically significant reductions in service utilization in most countries. The magnitudes of the cumulative shortfalls vary substantially by country, type of service, and time. The largest disruptions, on average, are estimated for outpatient consultations—a proxy for general healthcare utilization. Smaller cumulative shortfalls in the number of children receiving the third dose of the pentavalent vaccine are detected in most countries. The identified disruptions between March 2020 and June 2021 were associated with 113,962 additional deaths among women and children across 18 low- and middle-income countries.

This study was not designed to estimate the share of all-cause excess mortality caused by service disruptions. The number of indirect deaths we project is higher than the COVID-19 mortality officially reported by the 18 countries over the same time. Still, the officially reported number of deaths due directly to COVID-19 is grossly underreported in many countries [32]. Estimates of all-cause excess mortality published in May 2022 by WHO indicate a much greater level of mortality, with 597,422 excess deaths estimated to have occurred in the 18 countries by June 2021. No data were provided on the relative share of direct and indirect deaths within the total estimated number of excess deaths by country or globally. Regardless of the exact numbers, our findings illustrate that indirect deaths due to reductions in service coverage threaten to reverse gains in maternal and child mortality reduction achieved over a multi-year period before the COVID-19 outbreak. Yet, there is a clear need to strengthen country systems to track levels and trends in mortality by cause.

Service disruptions were largest during the first quarter of the pandemic, regardless of the timing of high reported COVID-19-related mortality or the stringency of policies imposed to contain the virus’ spread. This pattern may suggest a process of adaptation and learning. Individuals, health systems, and governments initially responded to the pandemic with uncertainty due to limited knowledge of the virus, transmission dynamics, risk, and safety measures. As more information became available, perceptions and behaviors might have changed. Alternatively, fatigue from mobility restrictions and social distancing could have influenced behavior patterns as the months elapsed. The duration of the pandemic may also have allowed time for health systems to adapt service provision, including combining multiple services in a single visit and transitioning care to the community level.

We also show a relationship between mobility restrictions and the magnitude of disruptions, highlighting the trade-offs inherent to the difficult policy decisions governments worldwide have had to make since the beginning of the pandemic. Imposing mobility and social gathering restrictions to contain the spread of the virus and protect those at high risk of COVID-19 mortality can come at the cost of reduced utilization of life-saving essential health services. In Nigeria, for example, a third of women surveyed during exit interviews after receiving RMNCH services reported not being able to access such services at some point since the start of the pandemic, with the most cited reasons being an inability to leave their household due to the lockdown, or because of the shutdowns and increased costs of public transportation [33]. These same issues were cited in other settings [33,34]. Even when the mobility restrictions do not specifically restrict health facility attendance, their introduction might affect individuals’ perceptions of whether services are available and the infection risk associated with visiting the health facilities. When such restrictions are imposed, the population’s ability to access essential health services must be maintained.

We estimate that the service disruptions were associated with increases in U5 and maternal mortality on the order of 2% to 5% for most countries in our analysis. The magnitude of the excess mortality is well below many scenarios presented at the onset of the pandemic, including that proposed by Roberton and colleagues, which hypothesized larger reductions in service utilization and predicted a relative increase in mortality of 10% [10]. However, the impact is proportional to the extent of the service disruptions in each country: Some countries experienced indirect mortality in the range of 5% to 15% of the expected mortality in the absence of the pandemic. The type of services that were disrupted is also important. Countries that saw larger declines in the proportion of women delivering at a facility were more likely to see larger increases in maternal mortality, linked to reductions in the parenteral administration of uterotonics, antibiotics, and anticonvulsant interventions. Countries with larger disruptions to outpatient utilization saw larger increases in child mortality, driven by estimated reductions in curative child health services such as antibiotics for pneumonia and neonatal sepsis, oral rehydration solution for dehydration due to diarrhea, and artemisinin-combination therapy for malaria. Although the primary driver of mortality is the magnitude and duration of service disruptions and the consequent reduction in coverage of interventions, other factors such as a country’s baseline coverage levels, baseline mortality rates, and cause-of-death structure were also important for country-specific mortality estimates.

Our study has several important limitations. Data derived from country health management information systems used in this study predominantly reflect the utilization patterns in the public sector, and the type of public facilities reporting data can vary between indicators and countries (detailed in Table 1). Theoretically, there could be a shift between public and private providers, which our analysis would not account for as changes in utilization. Additionally, data gaps can affect the completeness and quality of the HMIS data. We conduct a robustness check confirming that these changes in reporting patterns do not drive our findings.

The mortality estimates generated by LiST are limited by the accuracy of the input data, the set of health interventions considered in the analysis, and the assumptions made in linking the disruption in specific services to the overall coverage of interventions. For some countries, the baseline coverage inputs may be inaccurate due to the most recent DHS or MICS survey being conducted several years prior to 2020. However, given that we generated our pandemic coverage estimates relative to the baseline coverage estimates and given that our LiST results are predominantly driven by this relative change (and not the absolute value of the pandemic estimate or the counterfactual), this issue is likely to have had little effect on our results. The analysis does not account for differential changes in health-seeking behaviors by the risk group of patients. Relative reductions in healthcare-seeking behavior among low-risk patients may cause overestimations in the predicted mortality. In contrast, relative reductions in the ability to access healthcare by high-risk patients may result in underestimated mortality.

The indirect effects of the COVID-19 pandemic may have changed mortality through other pathways not considered by the LiST analysis. For example, reduced quality of care may lower the effectiveness of interventions in saving lives, social distancing is likely to have changed patterns in the transmission of other communicable diseases beyond COVID-19, and people’s behaviors more broadly may have changed disease incidence in ways we do not yet understand. We also do not consider effects such as malnutrition due to economic setbacks and disruptions to food markets. Other analyses have suggested that food insecurity could increase mortality by up to 10% in some countries [35]. Likewise, disruptions to family planning could affect birth outcomes and mortality rates for several years [12]. The selected services further represent an important but narrow set of services, and disruptions to chronic disease management, testing capacity, surgical services, and other life-saving health interventions are not considered. These broader effects are likely to be substantive and will result in cascading effects into the future.

Our findings have both short-term and long-term policy implications. Given the delays in COVID-19 vaccination access in low- and middle-income countries, the direct and indirect impacts of the COVID-19 crisis will likely persist in time. The prevalence of continued service disruptions, although to a lesser extent relative to the beginning of the pandemic, implies that children and mothers remain at higher risk of mortality during the protracted outbreak period. Continuity of essential health services during COVID-19 response must be monitored and maintained to minimize these preventable deaths. This study’s findings also highlight the need to invest in health system resiliency. Future studies should investigate the reasons why certain countries experienced more (or less) severe service disruptions by studying specific causes of disruptions and adaptations made by health facilities to address them. Together, these studies can inform future efforts to strengthen health systems to better prepare for and minimize loss of life during future health emergencies.

Additionally, this work helps understand how COVID-19 poses a profound threat to countries’ ability to progress toward UHC and SDGs (SDG 3.8.1). All 4 of the UHC service coverage index indicators for reproductive, maternal, newborn, and child health are represented in our analysis (i.e., family planning, ANC4, DTP3, and care-seeking for pneumonia). Given that most countries have experienced significant cumulative decreases in service volume in at least one of these 4 services since the start of the pandemic, our study provides evidence that COVID-19 is reversing longstanding progress toward achieving UHC by reducing coverage of essential health services.

## Conclusions

Service volume reported from health facilities across 18 low- and middle-income countries were disrupted for outpatient care and key reproductive, maternal, and child vaccination services during the pandemic. This use of facility data highlights the potential, with additional investment and validation, for these systems to play an important role in monitoring the resilience of health systems during times of shock. Substantial variation in the magnitude of disruption was identified during the pandemic and across services and countries. Overall, though the average disruption to maternal and child services was lower than many hypothesized scenarios as the pandemic’s onset, the decrease in intervention coverages is projected to be associated a substantial loss of life among women and children. These findings emphasize that safeguarding continuity of essential health services delivery must be maintained as part of the response to the COVID-19 pandemic and future crises particularly in low- and middle-income countries.

## Supporting information

S1 RECORD Checklist RECORD Checklist. (DOCX)

S1 Text Supporting Information. Table A. HMIS indicator definition and mapping. Table B. Sensitivity of disruption estimates between alternative definitions for deliveries. Table C. Sensitivity of disruption estimates between alternative definitions for outpatient consultations. Table D. Sensitivity of disruption estimates between alternative definitions for family planning. Table E. Linkage between service indicators to LiST interventions. Table F. Difference between expected and observed service coverage by month and country. Table G. Projections of mortality from LiST Model by Quarter. Table H. Bounding the mortality estimates using service disruption confidence intervals. Table I. Sensitivity analysis of linking decisions. Text A. Data notes. Fig A. Level of completeness by country and indicator. Table J. Cumulative change in service volume during the pandemic period (March 2020–June 2021) in a balanced panel of facilities. Table K. Sample size for balanced panel analysis (number of facilities). Table L. Data considerations. Table M. Percentage of reporting outliers by country in the prepandemic (January 2018–February 2020) and the pandemic (March 2020–June 2021) periods. Table N. Population totals and mortality rates references for analyzed countries. Fig B. Estimated and observed volume of additional indicators with officially reported COVID-19 deaths per 100,000 and mobility restrictions by country, January 2018–June 2021.
(DOCX)

## Abbreviations

BCG: bacillus Calmette–Guérin
COVID-19: Coronavirus Disease 2019
CSSE: Center for Systems Science and Engineering
DRC: Democratic Republic of the Congo
GFF: Global Financing Facility for Women, Children, and Adolescents
LiST: Lives Saved Tool
OPD: outpatient consultation
RMNCH: reproductive, maternal, and child
SDG: Sustainable Development Goal
UHC: Universal Health Coverage
WHO: World Health Organization
