# Supplementary material for: Healthcare utilization and maternal and child mortality during the COVID-19 pandemic in 18 low- and middle-income countries: An interrupted time-series analysis with mathematical modeling of administrative data
Source: PLoS Med. 2022 Aug 30;19(8):e1004070. doi: 10.1371/journal.pmed.1004070 (PMC9426906; doi:10.1371/journal.pmed.1004070)

# Appendix

**Table A.** **HMIS indicator definition and mapping**

|  | OPD | FP | ANC1 | ANC4 | DELIVERY | PNC1 | BCG | PENTA3 |
| --- | --- | --- | --- | --- | --- | --- | --- | --- |
| Afghanistan | Outpatient consultations | Family planning clients counseled | First antenatal care visits | Fourth antenatal care visits | Institutional deliveries | First postnatal visit | BCG doses administered | Penta 3 doses administered |
| Bangladesh | Total attendance  *or* Outpatient consultations | - | First antenatal care visits | Fourth antenatal care visits | Institutional deliveries *or* Normal deliveries | Number of mothers received PNC1 at facility | BCG doses given to children (0-11 months) | Penta 3 doses given to children (0-11m) |
| Cameroon | Outpatient consultations | - | Mothers registered for ANC | Fourth antenatal care visits | Institutional deliveries | Couples receiving PNC1 within 48 hours | - | Penta 3 doses given to children (0-11m) |
| DRC | New outpatient visits | - | First antenatal care visits | Fourth antenatal care visits | Skilled deliveries | PNC visit within 6 hours | - | Penta 3 doses administered |
| Ethiopia | Outpatient consultations | Family planning clients counseled | First antenatal care visits | Fourth antenatal care visits | Skilled deliveries | PNC visit within 7 days | - | Children given Penta 3 |
| Ghana | Outpatient consultations | - | Mothers registered for ANC | Fourth antenatal care visits | Normal deliveries | PNC visit within 48 hours | BCG doses administered | Children given Penta 3 |
| Guinea | Outpatient consultations | Family planning clients counseled | First antenatal care visits | Fourth antenatal care visits | Institutional deliveries by skilled birth attendants | - | BCG doses administered | Penta 3 doses administered |
| Haiti | New outpatient visits | Family planning clients counseled | First antenatal care visits during the first trimester | Fourth antenatal care visits | Institutional deliveries | First postnatal visit | BCG doses administered | Penta 3 doses administered |
| Kenya | Total attendance | Family planning clients counseled | First antenatal care visits | Fourth antenatal care visits | Institutional deliveries | First postnatal visit | - | Penta 3 doses administered |
| Liberia | New outpatient visits | Family planning clients counseled | First antenatal care visits | Fourth antenatal care visits | Institutional deliveries by skilled birth attendants | First postnatal visit | BCG doses administered | Penta 3 doses administered |
| Madagascar | New outpatient visits | Family planning clients counseled | First antenatal care visits | Fourth antenatal care visits | Total deliveries | PNC visit within 6 days | BCG doses given to children (0-11 months) | Penta 3 doses given to children (0-11m) |
| Malawi | Total attendance | Family planning clients counseled | First antenatal care visits | Fourth antenatal care visits | Institutional deliveries | Baby checked within 2 days | BCG doses given to children (0-11 months) | Penta 3 doses given to children (0-11m) |
| Mali | Outpatient consultations | Family planning clients counseled | - | - | Institutional deliveries by skilled birth attendants | First postnatal visit | BCG doses given to children (0-11 months) | Penta 3 doses administered |
| Nigeria | Total attendance | Family planning clients counseled | First antenatal care visits | Fourth antenatal care visits | Total deliveries | Total PNC visits | BCG doses administered | Penta 3 doses administered |
| Senegal | Outpatient consultations | - | First antenatal care visits | Fourth antenatal care visits | Facility deliveries | Mothers received PNC1 | BCG doses given to children (0-11 months) | Penta 3 doses given to children (0-11m) |
| Sierra Leone | Total attendance | Family planning clients counseled | First antenatal care visits | Fourth antenatal care visits | Normal deliveries | Postnatal visit 1st contact | BCG doses administered | Penta 3 doses administered |
| Somalia | New outpatient visits | - | First antenatal care visits | Fourth antenatal care visits | Normal deliveries | PNC visit within 48 hours | BCG doses administered | Penta 3 doses administered |
| Uganda | New outpatient visits | - | First antenatal care visits | Fourth antenatal care visits | Institutional deliveries | - | BCG doses administered | Penta 3 doses administered |

Note: DRC is Democratic Republic of the Congo. ANC1 refers to First Antenatal Care Visit. ANC4 refers to the Fourth Antenatal Care Visit. BCG refers to Bacillus Calmette–Guérin vaccination. FP refers to Family Planning Consultations. OPD refers to Outpatient visits. Penta3 refers to the Third dose of Pentavalent vaccine. PNC1 refers to First Postnatal Care Visit.

**Table B. Sensitivity of disruption estimates between alternative definitions for deliveries**

| Country | Indicator definitions | Cumulative shortfall | Lower 95% CI | Upper 95% CI |
| --- | --- | --- | --- | --- |
| Guinea | Skilled deliveries within a hospital | -5.0% | -10.3% | 0.3% |
|  | Total hospital deliveries | -7.0% | -11.5% | -2.6% |
|  | Skilled deliveries within a health center | 0.4% | -4.1% | 5.0% |
|  | Total health center deliveries | 0.8% | -3.8% | 5.5% |
|  | Skilled deliveries within a health post | -3.0% | -10.0% | 3.9% |
|  | Total deliveries within a health post | -3.9% | -11.8% | 4.0% |
| Mali | Skilled deliveries in a health center | -3.2% | -7.2% | 0.8% |
|  | Total deliveries within a health center | -3.8% | -5.4% | -2.2% |
| Malawi | Total institutional deliveries | -3.6% | -6.7% | -0.5% |
|  | Skilled institutional deliveries | -4.0% | -7.1% | -0.8% |
| DRC | Total deliveries | -0.2% | -1.3% | 0.8% |
|  | Total skilled deliveries | -0.3% | -1.3% | 0.8% |

**Table C. Sensitivity of disruption estimates between alternative definitions for outpatient consultations**

| Country | Indicator definitions | Cumulative shortfall | Lower 95% CI | Upper 95% CI |
| --- | --- | --- | --- | --- |
| Bangladesh | Inpatient admissions | -41.6% | -46.4% | -36.8% |
|  | Outpatient admissions | -40.0% | -44.7% | -35.4% |
| Liberia | Headcount | -6.7% | -13.7% | 0.3% |
|  | Adult outpatient consultations | -7.4% | -16.1% | 1.2% |
| Sierra Leone | Headcount | -6.8% | -20.0% | 6.3% |
|  | Outpatient consultations | -14.8% | -37.3% | 7.6% |

**Table D. Sensitivity of disruption estimates between alternative definitions for family planning**

| Country | Indicator definitions | Cumulative shortfall | Lower 95% CI | Upper 95% CI |
| --- | --- | --- | --- | --- |
| Afghanistan | Family planning consultations in health posts | 1.90% | -0.90% | 4.70% |
|  | Cycles of oral pills distributed at health posts | 14.90% | -5.30% | 35.10% |
|  | Cycles of oral pills distributed at health centers | -11.40% | -34.80% | 12.00% |
| Guinea | Family planning consultations | -12.30% | -20.20% | -4.50% |
|  | SNIS Quantités vendues d'ovrette (plaquettes) | 0.30% | -15.20% | 15.80% |
| Haiti | Nombre total de Femmes Utilisateurs PP | -6.60% | -30.40% | 17.10% |
|  | Family planning consultations | -3.00% | -10.10% | 4.10% |
| Liberia | Family planning consultations | -2.90% | -16.00% | 10.30% |
|  | Oral contraceptives users | 5.30% | -7.10% | 17.70% |
| Mali | Oral contraceptives users | -8.50% | -33.50% | 16.50% |
|  | Family planning consultations | -2.50% | -38.60% | 33.60% |
| Malawi | Oral contraceptives users | -4.80% | -17.00% | 7.40% |
|  | New family planning consultations | 2.50% | -14.00% | 19.10% |
|  | Returning family planning consultations | 5.40% | -2.10% | 12.80% |
| DRC | New family planning consultations | 9.10% | 5.50% | 12.70% |
|  | Returning family planning consultations | 21.70% | 14.80% | 28.50% |
|  | Oral contraceptives users | 28.10% | 14.80% | 41.30% |

**Table E.** **Linkage between service indicators to LiST interventions**

| **Service indicator** | **LiST intervention** |
| --- | --- |
| ANC 4 | Intermittent preventive treatment of malaria during pregnancy Calcium supplementation Micronutrient supplementation (iron and multiple micronutrients) Iron supplementation in pregnancy Multiple micronutrient supplementation in pregnancy Balanced energy supplementation Hypertensive disorder case management Diabetes case management Malaria case management |
| ANC 1 (or ANC 4 when ANC 1 not available) | Folic acid supplementation/fortification Blanket iron supplementation/fortification Tetanus toxoid vaccination Syphilis detection and treatment |
| Facility delivery | Post abortion case management Ectopic pregnancy case management Thermal protection Clean cord care Clean birth environment Immediate drying and additional stimulation Neonatal resuscitation Antenatal corticosteroids for preterm labor Antibiotics for preterm or prolonged PROM Parenteral administration of anti-convulsants Parenteral administration of uterotonics Parenteral administration of antibiotics Assisted vaginal delivery Manual removal of placenta Removal of retained products of conception Surgery Blood transfusion Induction of labor for pregnancies lasting 41+ weeks Prevalence of early initiation of breastfeeding Safe abortion services |
| PNC 1 (or Facility delivery when PNC 1 not available) | Complementary feeding - education only Complementary feeding - supplementary feeding and education |
| PNC 1 (or OPD when PNC 1 not available) | Maternal sepsis case management Kangaroo mother care Full supportive care for prematurity Full supportive care for neonatal sepsis/pneumonia |
| OPD | Case management of neonatal sepsis/pneumonia Oral antibiotics for neonatal sepsis Injectable antibiotics for neonatal sepsis Oral rehydration solution Antibiotics for treatment of dysentery Zinc for treatment of diarrhea Oral antibiotics for pneumonia Vitamin A for treatment of measles Artemisinin compounds for treatment of malaria SAM - treatment for severe acute malnutrition MAM - treatment for moderate acute malnutrition |
| Penta 3 | Polio vaccine Pentavalent vaccine DPT vaccine H. influenzae b vaccine HepB vaccine Pneumococcal vaccine Rotavirus vaccine Meningococcal A Malaria vaccine Measles vaccine Vitamin A supplementation Zinc supplementation |
| BCG (or Penta 3 when BCG not available) | BCG vaccine |

**Table F. Difference between expected and observed service coverage by month and country**

Note: This table contains pre-censored estimates. Results from indicators with poor completeness are not reported in the primary findings.

|  | Country | Mar 2020 | Apr 2020 | May 2020 | Jun 2020 | Jul 2020 | Aug 2020 | Sep 2020 | Oct 2020 | Nov 2020 | Dec 2020 | Jan 2021 | Feb 2021 | Mar 2021 | Apr 2021 | May 2021 | Jun 2021 |
| --- | --- | --- | --- | --- | --- | --- | --- | --- | --- | --- | --- | --- | --- | --- | --- | --- | --- |
| OPD | Afghanistan | -3.5% | -19.7% | -22.4% | -10.4% | -6.2% | -7.5% | 1.0% | -5.1% | -5.4% | -7.7% | -3.3% | 5.2% | -3.5% | -1.9% | -12.5% | 15.7% |
|  | Bangladesh | -21.4% | -74.3% | -73.1% | -55.5% | -59.4% | -45.7% | -32.9% | -30.0% | -19.2% | -19.5% | -22.7% | -25.2% | -23.8% | -42.8% | -44.1% | -22.8% |
|  | Cameroon | -3.5% | -10.8% | -6.6% | 0.4% | -5.0% | 0.1% | -1.9% | -5.9% | -3.0% | 4.8% | 10.2% | 13.1% | 6.4% | 0.0% | -1.8% | 4.0% |
|  | DRC | -4.2% | -6.4% | -4.7% | -3.2% | -4.6% | -4.4% | -3.3% | -5.0% | -5.0% | -6.4% | -8.4% | -9.0% | -9.5% | -9.6% | -7.9% | -10.7% |
|  | Ethiopia | -9.4% | -24.3% | -19.0% | -16.7% | -18.0% | -16.6% | -15.2% | -12.8% | -12.9% | -10.2% | -10.0% | -7.5% | -8.4% | -8.5% |  |  |
|  | Ghana | -9.4% | -28.3% | -29.3% | -23.0% | -21.5% | -16.7% | -19.0% | -18.5% | -9.3% | 1.8% | -6.7% | -6.7% | -4.7% | -5.4% | -10.4% | -4.4% |
|  | Guinea | -8.5% | -23.9% | -27.6% | -0.8% | -14.4% | -6.8% | -15.4% | -18.2% | -14.6% | -11.1% | -20.5% | -17.9% | -15.3% | -17.5% | -22.0% | -4.0% |
|  | Haiti | -12.8% | -40.7% | -43.5% | -41.2% | -32.3% | -23.2% | -5.1% | -16.9% | -22.0% | -17.7% | -21.9% | -27.9% | -21.7% | -29.0% | -29.2% | -26.6% |
|  | Kenya | -3.5% | -30.7% | -36.9% | -26.8% | -31.3% | -23.4% | -23.6% | -21.5% | -8.7% | -33.3% | -36.5% | -16.0% | -3.3% | -19.9% | -23.0% | -13.0% |
|  | Liberia | -7.5% | -29.0% | -20.3% | 15.4% | -1.4% | 1.0% | 3.5% | -11.9% | -3.4% | 21.6% | 7.5% | 14.1% | 22.8% | 9.8% | 1.2% | 22.4% |
|  | Madagascar | -6.1% | -12.4% | -5.0% | -7.3% | -17.8% | -24.6% | -15.1% | -11.1% | -1.1% | 1.5% | -2.2% | -9.2% | 0.8% | 4.3% | -0.7% | -3.2% |
|  | Malawi | 14.6% | -11.6% | -17.4% | -12.0% | -19.3% | -17.1% | -5.4% | -11.6% | 5.6% | 6.5% | -6.1% | -16.0% | -1.2% | -8.4% | -13.4% | 6.2% |
|  | Mali | 2.6% | -9.2% | -14.0% | -5.2% | -6.3% | 4.5% | -1.6% | -1.5% | -9.2% | -5.7% | -5.1% | -10.8% | -2.3% | 0.8% | -7.7% | -2.2% |
|  | Nigeria | -1.1% | -15.6% | -23.9% | -13.3% | -22.3% | -16.6% | -15.7% | -15.1% | -12.8% | -7.9% | -10.9% | -14.8% | -13.6% | -15.8% | -30.7% |  |
|  | Senegal | -2.4% | -26.0% | -36.4% | -11.4% | -12.8% | 8.5% | -19.9% | 13.1% | 11.0% | 6.2% | -6.9% | -7.8% | -0.3% | -2.2% | -12.8% | 10.3% |
|  | Sierra Leone | -7.5% | -15.7% | -13.2% | -3.4% | -9.4% | -10.6% | -15.7% | -21.9% | -19.2% | -16.5% | -13.7% | -18.0% |  | -11.5% | -18.8% |  |
|  | Somalia | 0.9% | -2.0% | -12.0% | 7.6% | 3.0% | 12.6% | 13.2% | 9.5% | 11.6% | 7.5% | 9.2% | 8.1% | 7.7% | 3.5% | 9.0% | 20.8% |
|  | Uganda | -6.4% | -27.6% | -25.2% | -17.9% | -24.1% | -19.2% | -15.4% | -17.3% | -13.7% | -7.5% | -24.0% | -16.4% | -19.4% | -25.8% | -19.1% | -20.5% |
|  | Total | -4.9% | -22.7% | -23.9% | -12.5% | -16.8% | -11.4% | -10.4% | -11.2% | -7.3% | -5.2% | -9.6% | -9.0% | -5.3% | -10.0% | -14.3% | -1.9% |
| FAMILY PLANNING | Afghanistan | -0.8% | -16.7% | -18.5% | -14.1% | -12.9% | -10.6% | -6.6% | 0.4% | -1.4% | -1.7% | 19.7% | 18.5% | 0.5% | -0.7% | -11.8% | 0.6% |
|  | Ethiopia | -3.2% | -4.3% | 2.9% | 2.5% | 0.9% | 5.6% | 3.2% | -0.1% | 2.9% | 5.1% |  |  |  |  |  |  |
|  | Guinea | -4.8% | -13.3% | -8.7% | -1.9% | -6.5% | -7.4% | -11.8% | -20.3% | -13.4% | -12.8% | -10.0% | -9.9% | -12.3% | -9.5% | -9.7% | -3.9% |
|  | Haiti | -4.1% | -7.1% | -7.2% | -2.4% | -1.4% | 2.2% | 5.9% | 6.7% | 6.4% | 6.2% | -2.7% | 0.7% | 0.3% | -2.7% | -4.8% | -2.5% |
|  | Kenya | 1.7% | -5.4% | -1.9% | 12.0% | 6.4% | 4.6% | 8.3% | -0.3% | 1.3% | -23.9% | -20.2% | -3.0% | 5.4% | -6.6% | -10.0% | -7.6% |
|  | Liberia | -3.7% | -3.6% | 21.5% | 26.2% | -7.3% | 0.6% | -4.0% | -11.1% | -14.8% | -12.0% | -30.4% | -34.5% | -12.3% | -12.0% | -14.0% | 2.1% |
|  | Madagascar | -4.5% | -6.3% | -5.3% | -3.0% | -5.9% | -4.0% | 0.9% | -4.0% | 2.3% | -5.6% | -15.4% | -20.4% | -17.2% | -22.6% | -27.5% | -5.2% |
|  | Malawi | 2.0% | -5.2% | 0.6% | 2.2% | 5.5% | 5.9% | 16.5% | 1.8% | 11.3% | 20.4% | 2.7% | -0.3% | 14.7% | 8.4% | 3.4% | 8.8% |
|  | Mali | -15.0% | -15.6% | -22.6% | -11.7% | -24.2% | -19.3% | -9.6% | 4.2% | -17.8% | -27.7% | -23.5% | -16.1% | -25.4% | -21.2% | -30.7% | -21.5% |
|  | Nigeria | -0.6% | -12.1% | -21.3% | 0.1% | -9.4% | 1.7% | 3.6% | -1.6% | -3.3% | -2.9% | -9.3% | -6.8% | -9.8% | -11.7% | -15.8% |  |
|  | Sierra Leone | -3.9% | -5.5% | -8.9% | -4.3% | -6.0% | -9.0% | -32.3% | -37.3% | -39.1% | -35.9% | -35.4% | -42.5% |  | -38.2% | -41.5% |  |
|  | Total | -3.5% | -8.6% | -6.6% | 1.1% | -5.6% | -2.5% | -2.1% | -5.3% | -5.4% | -7.2% | -11.5% | -10.1% | -5.0% | -10.4% | -14.7% | -1.9% |

| ANC1 | Afghanistan | -7.6% | -23.5% | -19.9% | -4.4% | -6.1% | -6.1% | 3.0% | -4.4% | -7.3% | -8.9% | -7.1% | -2.5% | -13.0% | -11.0% | -22.0% | 5.8% |
| --- | --- | --- | --- | --- | --- | --- | --- | --- | --- | --- | --- | --- | --- | --- | --- | --- | --- |
|  | Bangladesh | -16.3% | -49.9% | -41.7% | -29.4% | -27.5% | -24.8% | -8.5% | -8.4% | -11.2% | -10.8% | -11.5% | -12.3% | -11.1% | -23.8% | -19.3% | -7.5% |
|  | Cameroon | 3.3% | 0.2% | -3.9% | 0.1% | -4.5% | 7.2% | 3.1% | 1.4% | 8.5% | 17.4% | 5.1% | 14.0% | 17.2% | 10.3% | 14.9% | 20.5% |
|  | DRC | 0.2% | 1.4% | 0.7% | 3.9% | 1.9% | 0.7% | 3.8% | 1.1% | 0.4% | 3.1% | 1.1% | 2.1% | 2.7% | 2.5% | 4.6% | 5.6% |
|  | Ethiopia | -10.2% | -16.3% | 2.3% | -1.2% | -8.3% | 1.1% | 0.7% | -0.1% | -0.7% | 4.2% | -1.2% | -4.3% | -4.1% | -0.6% |  |  |
|  | Ghana | 2.6% | -1.8% | -4.0% | 19.4% | 1.0% | 3.4% | 5.3% | 4.3% | 9.0% | 19.0% | -3.3% | 7.8% | 13.7% | 5.1% | -0.4% | 14.9% |
|  | Guinea | 1.2% | -9.3% | -6.7% | 11.8% | -2.9% | 4.7% | 1.2% | -4.1% | 6.1% | 1.9% | 7.0% | 6.0% | 9.1% | 5.4% | 4.9% | 11.7% |
|  | Haiti | -2.4% | -19.9% | -17.2% | -8.1% | -10.2% | -5.3% | 16.2% | 0.7% | -1.1% | 0.3% | 2.5% | 18.1% | 19.3% | 2.7% | 7.9% | 17.7% |
|  | Kenya | -2.7% | -5.1% | -2.6% | 12.1% | -3.5% | -0.4% | 3.8% | -3.8% | 3.9% | -20.4% | -11.8% | 18.0% | 19.9% | 6.3% | -4.1% | 8.8% |
|  | Liberia | -7.2% | -17.6% | -6.5% | 10.3% | -6.9% | -2.9% | -3.5% | -13.7% | -3.2% | 12.9% | -5.3% | -3.8% | -2.9% | -10.5% | -12.4% | 0.1% |
|  | Madagascar | -4.0% | -9.3% | -1.0% | 14.1% | -3.7% | -1.8% | 11.3% | -4.9% | 6.3% | 1.9% |  |  |  |  |  |  |
|  | Malawi | 6.5% | -0.5% | -6.8% | 3.3% | -10.1% | -4.0% | 20.0% | -17.1% | -10.3% | 1.1% | -13.5% | -8.0% | -6.1% | -15.7% | -9.0% | -4.9% |
|  | Nigeria | -1.1% | -14.9% | -21.2% | 14.7% | -16.1% | 2.1% | -1.7% | -4.0% | -0.1% | -3.4% | -13.5% | -10.7% | -3.8% | -16.8% | -9.5% |  |
|  | Senegal | 3.3% | -2.5% | -20.7% | 11.0% | -28.1% | 0.7% | -16.1% | -18.7% | -10.5% | -10.7% | -19.3% | -13.2% | 0.7% | 0.3% | -13.3% | 9.2% |
|  | Sierra Leone | -1.4% | -6.8% | -12.7% | 8.2% | -4.5% | -4.7% | 3.5% | -3.2% | -2.7% | 9.8% | 1.2% | 0.9% |  | 5.2% | -9.1% |  |
|  | Somalia | -2.9% | -7.2% | -12.2% | 4.9% | -1.5% | 9.4% | 7.8% | 6.7% | 6.3% | 8.6% | 10.5% | 14.8% | 11.2% | 3.7% | 8.0% | 19.9% |
|  | Uganda | 0.1% | -10.1% | -6.4% | 12.6% | -6.2% | -0.8% | -2.7% | -5.9% | 2.2% | 12.2% | -12.0% | 5.4% | 13.3% | 0.5% | -6.6% | 6.7% |
|  | Total | -2.3% | -11.4% | -10.6% | 4.9% | -8.1% | -1.3% | 2.8% | -4.4% | -0.2% | 2.2% | -4.4% | 2.0% | 4.4% | -2.3% | -4.4% | 8.3% |
| ANC4 | Afghanistan | 3.0% | -8.2% | -12.7% | -9.0% | -11.5% | -5.5% | -2.6% | 3.4% | 2.7% | 1.6% | 3.5% | -2.4% | -3.2% | 0.0% | -5.1% | 3.7% |
|  | Bangladesh | -26.5% | -24.2% | -41.8% | -39.1% | -40.1% | -46.4% | -26.4% | -38.0% | -41.0% | -32.0% | -34.6% | -32.0% | -31.2% | -32.5% | -35.5% | -34.6% |
|  | Cameroon | -0.7% | -0.7% | -8.2% | -1.1% | -4.1% | -4.4% | -0.1% | -1.7% | 1.9% | 6.7% | -3.7% | 1.5% | 2.6% | 3.1% | 1.5% | 5.0% |
|  | DRC | 1.3% | 1.6% | 1.4% | 3.0% | 3.5% | 3.2% | 4.2% | 3.5% | 2.9% | 3.7% | 3.8% | 4.3% | 4.8% | 6.2% | 10.6% | 7.4% |
|  | Ethiopia | -4.8% | -9.3% | -2.4% | -4.5% | -6.1% | -2.4% | -0.6% | 1.4% | 0.4% | 4.9% | 3.1% | 0.7% | 2.2% | 1.7% |  |  |
|  | Ghana | 1.2% | -7.6% | -10.1% | 2.3% | 2.0% | 2.3% | 3.4% | 4.9% | 8.3% | 15.3% | 2.4% | 9.3% | 16.3% | 12.8% | 9.0% | 17.2% |
|  | Guinea | -4.4% | -13.6% | -11.5% | -6.3% | -10.7% | -8.2% | -9.0% | -9.7% | -5.7% | -7.0% | -6.8% | -8.9% | -12.7% | -14.2% | -15.9% | -14.9% |
|  | Haiti | -5.7% | -21.1% | -20.1% | -20.8% | -23.4% | -19.2% | -3.7% | -12.5% | -2.6% | 3.1% | 4.9% | 11.1% | 9.1% | 8.1% | 6.9% | 11.3% |
|  | Kenya | -6.4% | -13.9% | -21.7% | -14.1% | -14.2% | -13.3% | -5.5% | -10.6% | -7.0% | -25.9% | -9.5% | -14.3% | -12.7% | -13.7% | -10.3% | -1.2% |
|  | Liberia | -5.4% | -16.9% | -11.7% | 6.9% | 4.6% | -4.8% | -6.8% | -21.8% | -8.4% | 2.2% | -15.9% | -10.9% | -2.9% | -7.6% | -3.0% | 4.9% |
|  | Madagascar | 0.4% | -2.8% | -1.5% | 10.8% | 4.2% | 2.7% | 12.8% | 5.4% | 11.0% | 13.4% | 6.5% | 8.5% | 18.0% | 11.6% | 5.5% | 19.7% |
|  | Malawi | 20.6% | 7.9% | 6.8% | 21.7% | -4.8% | -4.2% | 14.1% | -13.8% | -6.6% | 9.1% | -4.0% | 5.1% | 20.6% | 4.1% | 14.1% | 16.0% |
|  | Nigeria | -10.4% | -15.2% | -27.4% | -5.0% | -17.4% | -7.9% | -9.4% | -17.0% | -16.1% | -15.6% | -28.6% | -26.9% | -24.2% | -28.3% | -28.0% |  |
|  | Senegal | -6.5% | -2.5% | -19.3% | -3.9% | -11.0% | 2.0% | 0.3% | -9.6% | -2.2% | -13.3% | -22.1% | -22.4% | -19.4% | -16.8% | -28.7% | -16.0% |
|  | Sierra Leone | -2.7% | -3.3% | -8.6% | -0.9% | -3.2% | -5.3% | 14.0% | 19.9% | 17.6% | 29.7% | 25.0% | 26.2% |  | 26.5% | 15.6% |  |
|  | Somalia | 4.8% | 0.5% | -6.6% | 20.2% | 5.8% | 24.7% | 19.5% | 15.3% | 19.0% | 29.3% | 33.8% | 39.1% | 30.6% | 18.2% | 23.1% | 34.8% |
|  | Uganda | -9.2% | -13.8% | -16.4% | -2.3% | -0.8% | -1.5% | 5.8% | 4.8% | 3.2% | 9.1% | 2.5% | 11.6% | 13.8% | 10.7% | 4.0% | 9.4% |
|  | Total | -3.0% | -8.3% | -12.5% | -1.8% | -7.7% | -4.4% | 0.6% | -4.4% | -1.2% | 1.8% | -2.3% | 0.1% | 0.9% | -0.3% | -2.5% | 5.3% |
| DELIVERY | Afghanistan | -6.5% | -11.2% | -12.4% | -15.2% | -12.8% | -10.3% | -6.8% | -8.5% | -7.9% | -7.7% | -11.3% | -13.3% | -13.4% | -9.2% | -14.0% | -15.6% |
|  | Bangladesh | -5.8% | -26.1% | -29.5% | -26.1% | -15.2% | -13.5% | -14.0% | -9.6% | -0.8% | 2.0% | -1.2% | -2.1% | -10.4% | -15.8% | -17.3% | -11.1% |
|  | Cameroon | 2.4% | 1.9% | 0.8% | 2.0% | 5.6% | 6.0% | 2.8% | 4.3% | 7.5% | 5.8% | 2.2% | 5.0% | 6.4% | 11.4% | 9.2% | 14.5% |
|  | DRC | -1.6% | -0.8% | 0.5% | 0.8% | 1.2% | -0.4% | -0.6% | -1.1% | -1.3% | -2.4% | -1.3% | -1.9% | -1.7% | -0.4% | 4.5% | 2.2% |
|  | Ethiopia | 1.4% | -1.2% | 2.9% | 2.7% | -1.1% | 0.9% | 1.2% | 4.8% | 6.6% | 8.9% | 9.0% | 7.8% | 7.9% | 7.8% |  |  |
|  | Ghana | 2.3% | -0.7% | 0.3% | -0.6% | 4.8% | -0.7% | -3.7% | -1.7% | 5.7% | -1.7% | 2.2% | 6.0% | 4.1% | 4.8% | 3.0% | 10.4% |
|  | Guinea | 3.4% | -3.1% | -0.5% | 2.9% | 0.8% | 2.9% | 1.1% | 1.9% | 4.4% | 0.1% | -0.8% | -0.2% | 1.6% | 3.0% | 5.8% | 8.5% |
|  | Haiti | -20.8% | -28.3% | -31.0% | -35.0% | -28.4% | -25.3% | -18.5% | -22.6% | -20.8% | -23.2% | -27.7% | -34.8% | -32.2% | -39.1% | -30.4% | -30.1% |
|  | Kenya | -0.6% | -4.0% | -3.9% | -0.5% | -1.5% | -1.3% | -1.2% | -4.0% | -2.5% | -13.9% | -5.5% | 0.4% | 3.0% | 3.7% | 0.6% | 6.2% |
|  | Liberia | 1.4% | -5.6% | -2.3% | 4.1% | -3.8% | -2.7% | -4.7% | -8.2% | -5.8% | -9.0% | -7.9% | -3.2% | -5.0% | -4.5% | -0.8% | 3.4% |
|  | Madagascar | 4.7% | 1.6% | 6.0% | 7.9% | 6.5% | 2.4% | 6.7% | 6.8% | 6.7% | 7.1% | 4.6% | 0.9% | 6.4% | 3.1% | 2.5% | 5.4% |
|  | Malawi | -0.7% | 0.4% | -1.0% | -4.7% | -4.3% | -3.9% | -9.1% | -9.0% | -4.0% | -5.3% | -3.9% | -7.2% | -8.5% | -9.7% | -8.0% | -9.1% |
|  | Mali | -7.2% | -4.0% | -7.9% | -5.6% | -5.5% | -2.1% | 2.1% | 1.9% | 1.6% | 3.8% | 1.5% | 4.7% | 0.1% | 12.2% | 0.6% | 9.6% |
|  | Nigeria | -0.3% | -5.4% | -13.1% | -6.3% | -10.6% | -11.2% | -8.9% | -7.9% | -6.9% | -10.3% | -13.3% | -14.5% | -13.8% | -10.2% | -13.4% |  |
|  | Senegal | -6.7% | -4.0% | -6.0% | -14.5% | -6.6% | -2.8% | -3.2% | -2.4% | -8.1% | -9.6% | -20.3% | -19.7% | -28.5% | -16.4% | -28.8% | -21.5% |
|  | Sierra Leone | -5.0% | -11.6% | -5.3% | -0.8% | 0.0% | 0.2% | -3.4% | -2.7% | -1.8% | -8.1% | -8.3% | -3.6% |  | 0.4% | 3.8% |  |
|  | Somalia | 4.1% | 0.9% | -1.4% | 1.8% | -0.2% | 0.3% | 2.8% | 1.7% | 2.3% | 3.1% | 5.9% | 10.0% | 12.8% | 3.9% | 4.9% | 8.0% |
|  | Uganda | -5.7% | -12.6% | -8.1% | -6.1% | -3.2% | -2.7% | -2.3% | -2.9% | 1.9% | -1.2% | -2.4% | 3.3% | 4.8% | 8.0% | 10.1% | 3.9% |
|  | Total | -2.3% | -6.3% | -6.2% | -5.2% | -4.1% | -3.6% | -3.3% | -3.3% | -1.3% | -3.4% | -4.4% | -3.5% | -3.9% | -2.6% | -4.0% | -1.0% |
| PNC1 | Afghanistan | -4.4% | -11.5% | -10.2% | -12.6% | -13.6% | -8.3% | -3.6% | -6.4% | -6.4% | -7.1% | -6.3% | -7.4% | -10.0% | -5.7% | -7.6% | -8.9% |
|  | Bangladesh | -16.1% | -29.4% | -27.1% | -26.5% | -26.0% | -16.4% | -16.3% | -13.8% | -15.6% | -11.8% | -14.3% | -12.8% | -16.7% | -17.8% | -20.0% | -14.0% |
|  | Cameroon | 3.6% | 0.9% | 2.5% | 4.8% | 8.2% | 10.8% | 5.9% | 6.9% | 11.8% | 10.6% | 6.7% | 10.1% | 12.7% | 15.7% | 15.9% | 18.6% |
|  | DRC | -1.9% | -1.4% | 0.1% | 0.5% | 0.4% | -0.8% | -1.1% | -0.5% | -1.7% | -2.7% | -1.3% | -1.9% | -2.4% | -1.0% | 3.8% | 1.6% |
|  | Ethiopia | 0.5% | -1.9% | 3.5% | 3.4% | -1.6% | 0.4% | -0.1% | 1.8% | 3.5% | 6.3% | 5.7% | 4.6% | 4.7% | 5.1% |  |  |
|  | Ghana | 2.9% | -0.7% | 1.0% | 4.4% | 5.5% | -0.5% | -1.0% | 2.7% | 7.7% | 2.6% | 3.8% | 6.1% | 7.5% | 7.4% | 4.9% | 13.1% |
|  | Haiti | -13.3% | -22.3% | -25.8% | -26.2% | -25.2% | -18.1% | -6.3% | -18.1% | -17.2% | -19.8% | -18.8% | -27.2% | -23.6% | -27.2% | -25.4% | -19.2% |
|  | Kenya | 1.9% | -5.1% | -5.6% | 1.4% | -3.9% | -3.9% | -0.7% | -10.4% | -9.4% | -9.4% | -7.2% | -8.2% | -1.8% | -3.9% | -10.6% | -4.6% |
|  | Liberia | 5.7% | -8.4% | -1.5% | -2.4% | -9.1% | -4.4% | -15.2% | -12.7% | -11.5% | -16.1% | -27.1% | -23.6% | -22.4% | -16.8% | -10.8% | -13.4% |
|  | Madagascar | 8.5% | 2.4% | 4.9% | 8.6% | 9.0% | 5.9% | 10.2% | 12.3% | 13.6% | 13.0% |  |  |  |  |  |  |
|  | Malawi | -0.1% | 4.0% | -4.3% | -2.9% | -5.4% | -2.4% | -9.7% | -3.5% | -6.8% | -6.5% | -6.7% | -7.0% | -5.2% | -8.9% | -9.7% | -13.9% |
|  | Mali | -1.8% | -0.9% | -3.5% | 1.0% | -3.2% | 2.1% | 3.5% | 3.7% | 5.7% | 2.5% | -0.9% | 7.5% | 5.7% | 17.5% | 2.9% | 11.5% |
|  | Nigeria | -2.7% | -10.1% | -17.3% | -8.3% | -12.6% | -3.0% | 12.8% | 11.4% | 18.3% | 22.3% | 19.7% | 28.1% | 30.3% | 30.8% | 29.9% |  |
|  | Senegal | -6.2% | -3.7% | -4.7% | -9.2% | -7.4% | 0.1% | 1.4% | -2.5% | -6.1% | -5.9% | -16.5% | -16.6% | -24.4% | -10.3% | -23.4% | -15.7% |
|  | Sierra Leone | -5.5% | -11.6% | -6.1% | 0.0% | -0.8% | 0.2% | -2.6% | -1.9% | -5.2% | -7.9% | -8.0% | -4.1% |  |  |  |  |
|  | Somalia | 1.9% | 3.3% | -0.2% | 16.6% | 5.5% | 12.9% | 14.1% | 15.3% | 19.2% | 13.9% | 19.7% | 27.0% | 21.2% | 19.1% | 14.2% | 28.5% |
|  | Total | -1.7% | -6.0% | -5.9% | -3.0% | -5.0% | -1.6% | -0.5% | -1.0% | 0.0% | -1.0% | -3.4% | -1.7% | -1.7% | 0.3% | -2.8% | -1.4% |
| BCG | Afghanistan | 7.2% | -5.4% | 0.1% | 5.1% | 2.1% | 5.7% | 10.4% | 4.0% | -0.1% | 5.7% | 8.1% | 10.0% | 4.6% | 8.0% | 0.5% | 19.3% |
|  | Bangladesh | -13.7% | -50.5% | -29.2% | 25.9% | 10.2% | 14.6% | 6.4% | 4.0% | -0.4% | -5.6% | 12.7% | 10.0% | 4.8% | -2.0% | -8.7% | 8.7% |
|  | Ghana | -2.5% | -3.5% | -8.0% | -6.3% | -2.2% | -2.1% | -5.2% | -3.7% | -1.7% | 1.2% | -2.9% | -0.5% | 0.0% | -1.8% | -5.5% | 2.8% |
|  | Guinea | -0.1% | -11.1% | -6.8% | 3.3% | -0.5% | -1.6% | -4.9% | -7.7% | 0.6% | -5.6% | 2.2% | 2.4% | 6.2% | 2.8% | 4.5% | 5.9% |
|  | Haiti | 10.1% | -22.0% | -19.6% | -10.8% | -2.3% | -10.5% | 13.9% | 1.3% | -20.7% | -28.0% | -45.8% | -45.4% | 28.7% | 28.6% | -11.2% | -10.3% |
|  | Liberia | 6.2% | -20.3% | 5.0% | 10.6% | -1.1% | 5.3% | 10.8% | -0.5% | 17.8% | 15.5% | 6.3% | 2.4% | 22.6% | 0.3% | 13.4% | 13.6% |
|  | Madagascar | 3.8% | -16.9% | -7.0% | 8.8% | -0.7% | -3.8% | 21.5% | -9.2% | 3.0% | 8.3% | -4.1% | -14.9% | -4.2% | -15.9% | -16.3% | 2.6% |
|  | Malawi | 2.8% | -5.1% | 0.7% | 1.7% | 1.6% | -0.2% | -3.9% | -5.6% | -1.4% | -1.4% | -7.5% | -7.1% | -4.3% | -1.4% | -7.7% | -3.7% |
|  | Mali | -4.8% | -21.3% | -14.3% | -0.8% | -14.9% | -3.8% | -4.2% | 1.7% | 2.2% | -8.8% | -28.9% | -15.3% | 4.1% | 3.0% | -10.1% | 10.5% |
|  | Nigeria | 1.5% | -6.8% | -12.9% | 4.0% | -5.9% | 1.0% | 6.1% | 5.3% | 9.3% | 18.9% | -2.4% | 3.2% | 4.2% | -1.5% | -3.1% |  |
|  | Senegal | -1.8% | 0.2% | 0.3% | 0.1% | -5.0% | 9.0% | 4.7% | -5.8% | -1.0% | -1.8% | -15.6% | -13.2% | -9.2% | -6.7% | -12.1% | -9.8% |
|  | Sierra Leone | -7.6% | -14.4% | -9.5% | 0.3% | -3.5% | -2.2% | -3.4% | -4.5% | -5.0% | -8.6% | -9.9% | -5.5% |  | -0.4% | 1.1% |  |
|  | Somalia | -2.1% | -4.8% | -13.5% | 3.2% | -0.7% | -8.2% | -3.7% | -0.3% | -6.9% | -0.8% | 8.3% | -0.6% | -0.9% | 1.2% | -6.3% | 6.3% |
|  | Uganda | -13.6% | -14.0% | -6.6% | -1.1% | -4.4% | -10.1% | -1.7% | -4.4% | -4.3% | 1.5% | -15.9% | -4.4% | -0.3% | -2.2% | -8.3% | -2.5% |
|  | Total | -1.0% | -14.0% | -8.7% | 3.1% | -1.9% | -0.5% | 3.3% | -1.8% | -0.6% | -0.7% | -6.8% | -5.6% | 4.3% | 0.9% | -5.0% | 3.6% |
| PENTA3 | Afghanistan | -3.7% | -15.5% | -13.7% | -0.1% | -2.5% | 0.9% | 5.2% | 1.9% | -2.4% | -0.9% | 3.1% | 2.0% | -6.0% | -2.6% | -10.6% | 16.9% |
|  | Bangladesh | -18.9% | -55.9% | -46.3% | -8.6% | 1.6% | 22.0% | 14.3% | 8.1% | 1.3% | -21.7% | -1.4% | 2.6% | 5.6% | -0.7% | -8.2% | 3.2% |
|  | Cameroon | -0.3% | -1.5% | -5.2% | 0.6% | 2.3% | -1.2% | 1.0% | -1.2% | -1.6% | 2.7% | -5.8% | -3.5% | -5.1% | -5.0% | -5.6% | 0.9% |
|  | DRC | -0.5% | -2.5% | -0.4% | -0.2% | 1.4% | -0.7% | 0.6% | 0.5% | -0.5% | 2.1% | 2.6% | 4.0% | 2.7% | -0.6% | 4.5% | 2.4% |
|  | Ethiopia | -6.0% | -7.6% | -0.7% | -0.9% | -0.9% | 0.7% | -1.7% | -3.3% | -3.8% | -0.7% | -1.8% | -3.9% | -5.8% | -4.8% |  |  |
|  | Ghana | -4.4% | -6.3% | -3.4% | 5.0% | 2.7% | 1.8% | -0.1% | 0.0% | 3.7% | 4.7% | 1.9% | 4.3% | 6.1% | 6.7% | 6.1% | 12.0% |
|  | Guinea | 0.5% | -12.9% | -9.4% | -3.3% | -1.7% | -5.4% | -4.9% | -7.1% | -2.8% | -7.6% | -2.4% | -1.6% | 0.5% | -1.5% | -2.3% | -1.3% |
|  | Haiti | 24.5% | -20.6% | -25.8% | -17.8% | 8.1% | 0.0% | 31.4% | 43.9% | 16.3% | 15.2% | -1.8% | -5.1% | 4.4% | 13.3% | 4.6% | -1.0% |
|  | Kenya | 1.7% | 1.9% | -3.6% | 9.1% | 1.9% | 0.5% | 4.6% | 0.1% | 3.9% | -5.3% | 1.2% | 4.5% | 13.4% | 14.4% | 9.3% | 17.9% |
|  | Liberia | -0.6% | -34.6% | -2.2% | -3.2% | 0.5% | 4.2% | 30.4% | 6.6% | 29.9% | -28.5% | -21.4% | 10.0% | 33.2% | -1.8% | 15.2% | 10.5% |
|  | Madagascar | -8.8% | -23.4% | -17.6% | 4.2% | -1.0% | -1.3% | 14.9% | -9.6% | 10.2% | 6.3% | -6.2% | -12.0% | -6.0% | -24.5% | -21.2% | 2.1% |
|  | Malawi | 2.0% | -9.4% | -2.9% | -2.0% | -2.3% | -1.7% | -4.2% | -5.4% | -4.6% | -5.4% | -10.7% | -14.0% | -11.6% | -12.7% | -11.6% | -9.6% |
|  | Mali | -9.0% | -26.6% | -23.5% | -7.1% | -16.6% | -5.8% | -8.8% | -8.0% | -5.4% | -10.2% | -10.5% | -7.3% | -8.3% | -3.1% | -16.7% | 3.3% |
|  | Nigeria | -4.1% | -12.9% | -18.4% | -1.5% | -7.6% | -3.9% | 3.1% | 5.9% | 6.5% | 14.6% | -4.4% | -0.6% | -1.8% | -8.4% | -10.9% |  |
|  | Senegal | -27.1% | -26.2% | -25.5% | -13.0% | -20.9% | -10.1% | -13.7% | -21.5% | -16.6% | -13.6% | -18.1% | -22.6% | -34.1% | -34.0% | -35.2% | -25.2% |
|  | Sierra Leone | -8.9% | -20.6% | -16.9% | -9.8% | -8.6% | -8.1% | -3.6% | -6.3% | -8.5% | -7.2% | -8.7% | -7.9% |  | -14.4% | -14.2% |  |
|  | Somalia | -4.5% | -8.7% | -12.4% | -0.5% | 0.6% | 2.2% | 5.7% | 0.4% | -1.1% | -4.0% | -2.1% | -0.8% | 3.6% | 0.0% | 3.2% | 8.2% |
|  | Uganda | -11.1% | -18.6% | -10.4% | 3.0% | 1.0% | -6.2% | -2.8% | 0.9% | -9.1% | 2.1% | -11.2% | -5.1% | 0.2% | -2.7% | -11.1% | -4.6% |
|  | Total | -0.044 | -0.168 | -0.132 | -0.026 | -0.023 | -0.007 | 0.04 | 0.003 | 0.009 | -0.032 | -0.054 | -0.032 | -0.005 | -0.046 | -0.062 | 0.024 |

Note: DRC is Democratic Republic of the Congo. ANC1 refers to First Antenatal Care Visit. ANC4 refers to the Fourth Antenatal Care Visit. BCG refers to Bacillus Calmette–Guérin vaccination. FP refers to Family Planning Consultations. OPD refers to Outpatient visits. Penta3 refers to the Third dose of Pentavalent vaccine. PNC1 refers to First Postnatal Care Visit.

**Table G. Projections of mortality from LiST Model by Quarter**

|  | Quarter |  | Child deaths (0-59 months) | |  | Neonatal deaths (< 1 month) | |  | Maternal deaths | |
| --- | --- | --- | --- | --- | --- | --- | --- | --- | --- | --- |
|  |  |  | Expected deaths | Additional deaths |  | Expected deaths | Additional deaths |  | Expected deaths | Additional deaths |
| Afghanistan | Q2 2020 |  | 18,724 | 1,204 |  | 11,235 | 673 |  | 1,933 | 97 |
|  | Q3 2020 |  | 18,724 | 456 |  | 11,235 | 339 |  | 1,933 | 74 |
|  | Q4 2020 |  | 18,724 | 507 |  | 11,235 | 334 |  | 1,933 | 58 |
|  | Q1 2021 |  | 19,029 | 415 |  | 11,448 | 400 |  | 1,968 | 94 |
|  | Q2 2021 |  | 19,029 | 384 |  | 11,448 | 404 |  | 1,968 | 96 |
| Bangladesh | Q2 2020 |  | 22,213 | 6,435 |  | 12,578 | 2,747 |  | 1,271 | 119 |
|  | Q3 2020 |  | 22,213 | 3,768 |  | 12,578 | 1,602 |  | 1,271 | 63 |
|  | Q4 2020 |  | 22,213 | 1,748 |  | 12,578 | 621 |  | 1,271 | 19 |
|  | Q1 2021 |  | 22,056 | 1,840 |  | 12,473 | 709 |  | 1,261 | 25 |
|  | Q2 2021 |  | 22,056 | 3,162 |  | 12,473 | 1,391 |  | 1,261 | 62 |
| Cameroon | Q2 2020 |  | 16,690 | 75 |  | 6,013 | 29 |  | 1,198 | -4 |
|  | Q3 2020 |  | 16,690 | -53 |  | 6,013 | -69 |  | 1,198 | -17 |
|  | Q4 2020 |  | 16,690 | -100 |  | 6,013 | -110 |  | 1,198 | -26 |
|  | Q1 2021 |  | 16,843 | -281 |  | 6,099 | -201 |  | 1,214 | -20 |
|  | Q2 2021 |  | 16,843 | -253 |  | 6,099 | -246 |  | 1,214 | -51 |
| DRC | Q2 2020 |  | 76,990 | 660 |  | 25,284 | 296 |  | 4,229 | -3 |
|  | Q3 2020 |  | 76,990 | 568 |  | 25,284 | 255 |  | 4,229 | -3 |
|  | Q4 2020 |  | 76,990 | 888 |  | 25,284 | 476 |  | 4,229 | 14 |
|  | Q1 2021 |  | 79,083 | 1,406 |  | 26,012 | 736 |  | 4,349 | 14 |
|  | Q2 2021 |  | 79,083 | 1,168 |  | 26,012 | 455 |  | 4,349 | -27 |
| Ethiopia | Q2 2020 |  | 49,493 | 1,202 |  | 25,560 | 387 |  | 3,637 | -1 |
|  | Q3 2020 |  | 49,493 | 953 |  | 25,560 | 294 |  | 3,637 | 1 |
|  | Q4 2020 |  | 49,493 | 423 |  | 25,560 | -70 |  | 3,637 | -31 |
|  | Q1 2021 |  | 50,008 | 332 |  | 25,805 | -41 |  | 3,656 | -35 |
|  | Q2 2021 |  | 50,008 | 91 |  | 25,805 | -34 |  | 3,656 | -11 |
| Ghana | Q2 2020 |  | 10,511 | 750 |  | 5,317 | 396 |  | 683 | 3 |
|  | Q3 2020 |  | 10,511 | 518 |  | 5,317 | 270 |  | 683 | -2 |
|  | Q4 2020 |  | 10,511 | 194 |  | 5,317 | 83 |  | 683 | -8 |
|  | Q1 2021 |  | 10,535 | 54 |  | 5,364 | -21 |  | 689 | -17 |
|  | Q2 2021 |  | 10,535 | 23 |  | 5,364 | -58 |  | 689 | -25 |
| Guinea | Q2 2020 |  | 11,519 | 414 |  | 3,651 | 101 |  | 675 | 5 |
|  | Q3 2020 |  | 11,519 | 267 |  | 3,651 | 52 |  | 675 | 1 |
|  | Q4 2020 |  | 11,519 | 318 |  | 3,651 | 59 |  | 675 | -1 |
|  | Q1 2021 |  | 11,757 | 402 |  | 3,755 | 94 |  | 693 | 4 |
|  | Q2 2021 |  | 11,757 | 274 |  | 3,755 | 21 |  | 693 | -5 |
| Haiti | Q2 2020 |  | 4,374 | 391 |  | 1,775 | 177 |  | 328 | 33 |
|  | Q3 2020 |  | 4,374 | 212 |  | 1,775 | 110 |  | 328 | 25 |
|  | Q4 2020 |  | 4,374 | 197 |  | 1,775 | 102 |  | 328 | 21 |
|  | Q1 2021 |  | 4,358 | 254 |  | 1,768 | 135 |  | 326 | 26 |
|  | Q2 2021 |  | 4,358 | 291 |  | 1,768 | 150 |  | 326 | 27 |
| Kenya | Q2 2020 |  | 14,917 | 1,177 |  | 7,357 | 437 |  | 1,287 | 18 |
|  | Q3 2020 |  | 14,917 | 954 |  | 7,357 | 340 |  | 1,287 | 9 |
|  | Q4 2020 |  | 14,917 | 938 |  | 7,357 | 436 |  | 1,287 | 37 |
|  | Q1 2021 |  | 15,059 | 653 |  | 7,470 | 216 |  | 1,306 | 6 |
|  | Q2 2021 |  | 15,059 | 560 |  | 7,470 | 128 |  | 1,306 | -14 |
| Liberia | Q2 2020 |  | 2,824 | 142 |  | 990 | 24 |  | 267 | 2 |
|  | Q3 2020 |  | 2,824 | -5 |  | 990 | 11 |  | 267 | 3 |
|  | Q4 2020 |  | 2,824 | -2 |  | 990 | 20 |  | 267 | 8 |
|  | Q1 2021 |  | 2,867 | -156 |  | 1,006 | -7 |  | 271 | 6 |
|  | Q2 2021 |  | 2,867 | -127 |  | 1,006 | -14 |  | 271 | 1 |
| Madagascar | Q2 2020 |  | 11,601 | 157 |  | 4,498 | -11 |  | 731 | -7 |
|  | Q3 2020 |  | 11,601 | 324 |  | 4,498 | 27 |  | 731 | -8 |
|  | Q4 2020 |  | 11,601 | 9 |  | 4,498 | -41 |  | 731 | -11 |
|  | Q1 2021 |  | 11,744 | 63 |  | 4,554 | -18 |  | 738 | -7 |
|  | Q2 2021 |  | 11,744 | 11 |  | 4,554 | -29 |  | 738 | -7 |
| Malawi | Q2 2020 |  | 7,492 | 446 |  | 3,543 | 217 |  | 552 | 6 |
|  | Q3 2020 |  | 7,492 | 538 |  | 3,543 | 307 |  | 552 | 27 |
|  | Q4 2020 |  | 7,492 | 137 |  | 3,543 | 133 |  | 552 | 31 |
|  | Q1 2021 |  | 7,614 | 397 |  | 3,612 | 254 |  | 563 | 31 |
|  | Q2 2021 |  | 7,614 | 370 |  | 3,612 | 271 |  | 563 | 42 |
| Mali | Q2 2020 |  | 19,321 | 611 |  | 6,647 | 303 |  | 1,142 | 28 |
|  | Q3 2020 |  | 19,321 | 104 |  | 6,647 | 65 |  | 1,142 | 8 |
|  | Q4 2020 |  | 19,321 | 204 |  | 6,647 | 25 |  | 1,142 | -10 |
|  | Q1 2021 |  | 19,793 | 233 |  | 6,837 | 34 |  | 1,173 | -10 |
|  | Q2 2021 |  | 19,793 | -58 |  | 6,837 | -161 |  | 1,173 | -36 |
| Nigeria | Q2 2020 |  | 222,905 | 14,126 |  | 67,788 | 2,281 |  | 17,230 | 381 |
|  | Q3 2020 |  | 222,905 | 14,128 |  | 67,788 | 2,524 |  | 17,230 | 415 |
|  | Q4 2020 |  | 222,905 | 8,691 |  | 67,788 | 1,873 |  | 17,230 | 385 |
|  | Q1 2021 |  | 225,551 | 10,922 |  | 69,084 | 2,911 |  | 17,530 | 657 |
|  | Q2 2021 |  | 225,551 | 12,066 |  | 69,084 | 2,187 |  | 17,530 | 395 |
| Senegal | Q2 2020 |  | 5,924 | 429 |  | 2,835 | 278 |  | 432 | 20 |
|  | Q3 2020 |  | 5,924 | 173 |  | 2,835 | 119 |  | 432 | 11 |
|  | Q4 2020 |  | 5,924 | -31 |  | 2,835 | 16 |  | 432 | 17 |
|  | Q1 2021 |  | 6,007 | 367 |  | 2,879 | 327 |  | 438 | 59 |
|  | Q2 2021 |  | 6,007 | 306 |  | 2,879 | 281 |  | 438 | 56 |
| Sierra Leone | Q2 2020 |  | 6,721 | 435 |  | 2,136 | 131 |  | 727 | 29 |
|  | Q3 2020 |  | 6,721 | 401 |  | 2,136 | 86 |  | 727 | 3 |
|  | Q4 2020 |  | 6,721 | 661 |  | 2,136 | 157 |  | 727 | 2 |
|  | Q1 2021 |  | 6,815 | 376 |  | 2,171 | 104 |  | 738 | 5 |
|  | Q2 2021 |  | 6,815 | 321 |  | 2,171 | 49 |  | 738 | -15 |
| Somalia | Q2 2020 |  | 19,709 | 52 |  | 6,236 | 34 |  | 1,375 | 0 |
|  | Q3 2020 |  | 19,709 | -131 |  | 6,236 | -47 |  | 1,375 | -1 |
|  | Q4 2020 |  | 19,709 | -146 |  | 6,236 | -64 |  | 1,375 | -2 |
|  | Q1 2021 |  | 20,226 | -191 |  | 6,418 | -117 |  | 1,413 | -6 |
|  | Q2 2021 |  | 20,226 | -197 |  | 6,418 | -98 |  | 1,413 | -4 |
| Uganda | Q2 2020 |  | 18,888 | 1,826 |  | 8,280 | 759 |  | 1,562 | 67 |
|  | Q3 2020 |  | 18,888 | 1,361 |  | 8,280 | 481 |  | 1,562 | 18 |
|  | Q4 2020 |  | 18,888 | 848 |  | 8,280 | 271 |  | 1,562 | 1 |
|  | Q1 2021 |  | 19,067 | 1,244 |  | 8,402 | 338 |  | 1,583 | -19 |
|  | Q2 2021 |  | 19,067 | 1,199 |  | 8,402 | 209 |  | 1,583 | -53 |

**Table H.** **Bounding the mortality estimates using service disruption confidence intervals**

|  | Additional deaths for March 2020 to June 2021 (child and maternal combined) | | | Relative difference from point estimate | |
| --- | --- | --- | --- | --- | --- |
|  | Point estimate | Using disruption estimate CI upper bound | Using disruption estimate CI lower bound | Lower bound mortality estimate | Upper bound mortality estimate |
| Afghanistan | 3,526 | 628 | 6,496 | -82.2% | 84.2% |
| Bangladesh | 17,878 | 10,687 | 25,460 | -40.2% | 42.4% |
| Cameroon | -731 | -1,677 | 231 | -- | -- |
| DRC | 4,928 | 1,850 | 8,028 | -62.5% | 62.9% |
| Ethiopia | 3,191 | 1,376 | 5,018 | -56.9% | 57.2% |
| Ghana | 1,556 | 725 | 2,401 | -53.4% | 54.3% |
| Guinea | 1,730 | -746 | 4,276 | -143.1% | 147.1% |
| Haiti | 1,534 | 965 | 2,114 | -37.1% | 37.8% |
| Kenya | 4,385 | 3,180 | 5,607 | -27.5% | 27.9% |
| Liberia | -100 | -887 | 701 | -- | -- |
| Madagascar | 560 | -437 | 1,569 | -178.0% | 180.3% |
| Malawi | 1,883 | -328 | 4,184 | -117.4% | 122.2% |
| Mali | 1,106 | -1,441 | 3,722 | -230.3% | 236.5% |
| Nigeria | 62,568 | 44,789 | 80,484 | -28.4% | 28.6% |
| Senegal | 1,453 | 743 | 2,180 | -48.8% | 50.1% |
| Sierra Leone | 2,328 | 1,184 | 3,502 | -49.1% | 50.4% |
| Somalia | -626 | -1,515 | 267 | -- | -- |
| Uganda | 6,703 | 4,317 | 9,129 | -35.6% | 36.2% |
| Total | 113,873 | 63,413 | 165,371 | -44.3% | 45.2% |

To calculate an upper bound of mortality, the input into the LiST model is the the upper limit of the 95% CI (confidence interval) from the cumulative disruption estimate is used for all the services. To calculate a lower bound, the lower limit of the 95% CI is used for all services. DRC is Democratic Republic of the Congo.

**Table I. Sensitivity analysis of linking decisions**

|  | Additional child deaths for Quarter 2, 2020 | | | | | | | | | Relative difference from the linking combination used for final analysis | | | | | | | |
| --- | --- | --- | --- | --- | --- | --- | --- | --- | --- | --- | --- | --- | --- | --- | --- | --- | --- |
|  | Linking combination used for final analysis | All LiST interventions linked to ANC 4 | All LiST interventions linked to Facility delivery | All LiST interventions linked to OPD | All LiST interventions linked to Penta 3 | Random linking (run 1) | Random linking (run 2) | Random linking (run 3) | Random linking (run 4) | All LiST interventions linked to ANC 4 | All LiST interventions linked to Facility delivery | All LiST interventions linked to OPD | All LiST interventions linked to Penta 3 | Random linking (run 1) | Random linking (run 2) | Random linking (run 3) | Random linking (run 4) |
| Afghanistan | 1,204 | 773 | 1,008 | 1,373 | 756 | 1,038 | 798 | 957 | 872 | -35.8% | -16.3% | 14.0% | -37.2% | -13.8% | -33.8% | -20.5% | -27.6% |
| Bangladesh | 6,435 | 4,249 | 3,245 | 8,816 | 4,492 | 4,661 | 4,068 | 4,577 | 4,805 | -34.0% | -49.6% | 37.0% | -30.2% | -27.6% | -36.8% | -28.9% | -25.3% |
| Cameroon | 75 | 131 | -59 | 220 | 78 | 89 | 111 | 82 | 96 | 75.2% | -179.8% | 194.7% | 5.0% | 18.9% | 48.9% | 10.3% | 28.4% |
| DRC | 660 | -450 | -36 | 1,095 | 231 | 148 | -192 | 142 | 53 | -168.2% | -105.5% | 65.9% | -65.1% | -77.6% | -129.1% | -78.5% | -91.9% |
| Ethiopia | 1,202 | 665 | -178 | 2,493 | 376 | 800 | 494 | 696 | 662 | -44.7% | -114.8% | 107.4% | -68.7% | -33.4% | -58.9% | -42.1% | -44.9% |
| Ghana | 750 | 279 | 17 | 1,568 | 85 | 463 | 298 | 433 | 351 | -62.8% | -97.7% | 109.1% | -88.7% | -38.3% | -60.3% | -42.3% | -53.2% |
| Guinea | 414 | 372 | 8 | 628 | 302 | 240 | 265 | 251 | 325 | -10.3% | -98.0% | 51.5% | -27.2% | -42.2% | -36.0% | -39.5% | -21.5% |
| Haiti | 391 | 214 | 329 | 442 | 222 | 344 | 242 | 296 | 242 | -45.2% | -15.9% | 13.0% | -43.2% | -11.9% | -38.0% | -24.4% | -38.1% |
| Kenya | 1,177 | 1,065 | 175 | 2,084 | -152 | 1,053 | 574 | 817 | 519 | -9.5% | -85.1% | 77.1% | -112.9% | -10.5% | -51.2% | -30.6% | -55.9% |
| Liberia | 142 | 111 | 19 | 173 | 206 | 73 | 150 | 101 | 160 | -22.0% | -86.6% | 22.0% | 44.7% | -48.6% | 5.7% | -29.1% | 12.5% |
| Madagascar | 157 | -68 | -160 | 259 | 390 | 73 | 110 | -12 | 131 | -143.4% | -201.8% | 64.9% | 147.9% | -53.6% | -30.1% | -107.4% | -16.9% |
| Malawi | 446 | -605 | 93 | 743 | 252 | 134 | -250 | 84 | 49 | -235.5% | -79.1% | 66.5% | -43.5% | -70.0% | -155.9% | -81.3% | -89.1% |
| Mali | 611 | 270 | 444 | 724 | 1,490 | 587 | 791 | 569 | 740 | -55.8% | -27.3% | 18.5% | 144.0% | -3.8% | 29.6% | -6.8% | 21.1% |
| Nigeria | 14,126 | 14,445 | 7,474 | 16,060 | 9,924 | 12,750 | 10,772 | 12,726 | 12,579 | 2.3% | -47.1% | 13.7% | -29.7% | -9.7% | -23.7% | -9.9% | -10.9% |
| Senegal | 429 | 222 | 212 | 671 | 583 | 385 | 316 | 393 | 449 | -48.2% | -50.6% | 56.4% | 35.9% | -10.4% | -26.4% | -8.5% | 4.6% |
| Sierra Leone | 435 | 188 | 261 | 484 | 718 | 361 | 369 | 307 | 490 | -56.9% | -40.1% | 11.1% | 64.9% | -17.0% | -15.2% | -29.5% | 12.7% |
| Somalia | 52 | -89 | -9 | 41 | 137 | -24 | 4 | -50 | -14 | -272.8% | -116.6% | -21.1% | 165.8% | -146.3% | -93.0% | -196.2% | -127.1% |
| Uganda | 1,826 | 1,063 | 876 | 2,380 | 848 | 1,346 | 1,201 | 1,195 | 1,261 | -41.8% | -52.0% | 30.3% | -53.6% | -26.3% | -34.2% | -34.6% | -30.9% |
| Total | 30,533 | 22,834 | 13,718 | 40,253 | 20,939 | 24,521 | 20,121 | 23,564 | 23,770 | -25.2% | -55.1% | 31.8% | -31.4% | -19.7% | -34.1% | -22.8% | -22.2% |

Note: The sensitivity analysis only focuses on the second quarter of 2020, given the disproportional contribution of service declines in the quarter to the total estimated mortality. DRC is Democratic Republic of the Congo. ANC4 refers to the Fourth Antenatal Care Visit. OPD refers to Outpatient visits. Penta3 refers to the Third dose of Pentavalent vaccine.

# Text A. Data notes

## **Service volume data**

The volume of services provided by health facilities is extracted directly from country Health Management Information Systems (HMIS). Service volume is self-reported by health facilities and is reviewed and validated with varying levels of rigor between countries and between specific indicators. Data were extracted at the facility-level for the eight indicators across all countries on August 22^nd^, 2021. When used for national level inference, these data have three sources of potential bias: data availability which affects the representativeness of the larger health sector, errors introduced through data recording, and shifts in reporting patterns which alter representativeness of data over time. These possible shortcomings can cause difficulties in interpretation of data, especially without detailed information on the patterns and contexts of the data collection systems.

Despite these challenges, the HMIS data are among the best sources to track changes in service coverage as they 1) contain data both before and after the COVID-19 pandemic, 2) contain data from a large number of facilities, indeed in many countries a near census of public facilities, and 3) are frequently updated. The interpretive challenges with this data, and the stability of results, with respect to data completeness, representativeness, and outlier values are explored below.

Data Completeness

Reporting patterns from health facilities may be affected by rollout or changes in data systems, or local conditions which prevent facilities from reporting. Bias is introduced when the trends in completeness are correlated with the change in service coverage. For instance, the level of completeness may depend on the amount of time given to enter reports into the system. In many countries, completeness looks better over time as late reports are submitted. A complete report is defined as a facility which reported a non-zero number of services, or reported a non-zero OPD for the same month, and have reported at least one during the calendar year. Facilities which do not report any services for the entire timeframe are not included. This completeness definition is driven wholly by HMIS reporting, rather the health facility master lists, which may be out of date. Figure K in this text illustrates the completeness of data for each indicator and country.

With consultation with and validation by with each ministry, periods of acute drops or trends in completeness, or indicators which were lower than 40% completeness, were identified and omitted from the dataset. After these drops, the final sample size for the analysis is presented in Table 1.

Overall, the completeness during the year of the pandemic for outpatient consultations is 2 percentage points greater than the pre-pandemic period and is therefore unlikely to affect the results. As a robustness check, we present results restricted to facilities with complete reporting and show that changes in completeness during the pandemic do not drive the findings.

Representativeness

In addition to reporting patterns from facilities within HMIS, there are segments of the health sector not captured in the HMIS. In most countries, the HMIS predominantly covers the public facilities. There are differences in the data availability by the level of the health system (hospitals, health centers, and lower-level facilities). This can be expected, as some health services are not provided at all levels of the health system, due to differences in the types indicators reported by the different levels, or because the HMIS system does not yet cover a specific facility type. In some countries, data are not available from specific regions of the country. These omissions and changes to address completeness issues are summarized in Table L of this text.

Identifying outliers

We identify and remove errors that result in outlier values, defined as observations within a facility-service group more than 10 deviations above the positive median absolute deviation (MAD), or,

$$Outlier>10*\frac{\left| X_{i}-\overline{X} \right|}{\mathrm{Median}\left( \left| X_{p}-\overline{X} \right| \right)}$$

Where $X_{p}$ are the observations above the median, $\overline{X}$ is the average value, and $X_{i}$ is each observation. The threshold of 10 was set with the goal of developing a conservative rule which would identify egregious data errors but less likely to overcorrect for events such as mass immunization campaigns. The proportion of total values identified as outliers during the pre-pandemic and post-pandemic periods is given in Table K of this text. The egregious outlier rate is low, often far below 1% for each country-indicator pair, and not appreciably different before and during the pandemic period. The code using for outlier detection is below, where uid is the facility identifier, indicatortype is the indicator name, and indicatorval is the service volume.

bys uid indicatortype: egen med = median(indicatorval) if !missing(indicatorval)

bys uid indicatortype: egen mad = mad(indicatorval) if indicatorval >= med & !missing(indicatorval)

gen mad_resid = abs(indicatorval - med)/mad

gen outlier = 0

replace outlier = 1 if mad_resid > 10 & !missing(mad_resid)

Sample code for analysis:

The code used for disruption estimation. The data structure is long format for each country, with one row for each facility-indicator-date triplet. The macro intvars contains the list of indicators for the country. The cluster variable districtdummy contains the unique numeric for the second administrative level. The covariates in the regression are indicatorval representing the service volume, date representing the months since the start of the pandemic in a continuous variable, the calendar month in a factor variable to account for seasonality, and postpandemic which is a binary variable representing whether the date was a postpandemic or prepandemic month. For monthly disruptions, the postpandemic term was replace by a nominal variable for each month of the pandemic.

foreach indicatortype in `intvars' {

xtreg indicatorval c.date i.month postpandemic if indicatortype == "`indicatortype'", fe cluster(districtdummy)

predict expect if indicatortype == "`indicatortype'" & !missing(indicatorval), xbu

replace predict = (expect - (_b[postpandemic]*postpandemic)) if indicatortype == "`indicatortype'"

gen diff = indicatorval-predict if indicatortype == "`indicatortype'"

egen diffmean_total = mean(diff) if indicatortype == "`indicatortype'" & postpandemic

egen predictmean_total = mean(predict) if indicatortype == "`indicatortype'" & postpandemic

replace b_shortfall = diffmean_total /predictmean_total if postpandemic & indicatortype == "`indicatortype'"

replace p_shortfall = 2*ttail(e(df_r),abs(_b[postpandemic]/_se[postpandemic])) if indicatortype == "`indicatortype'"

replace se_shortfall = _se[postpandemic] if indicatortype == "`indicatortype'"

}

**Fig A.** Level of completeness by country and indicator


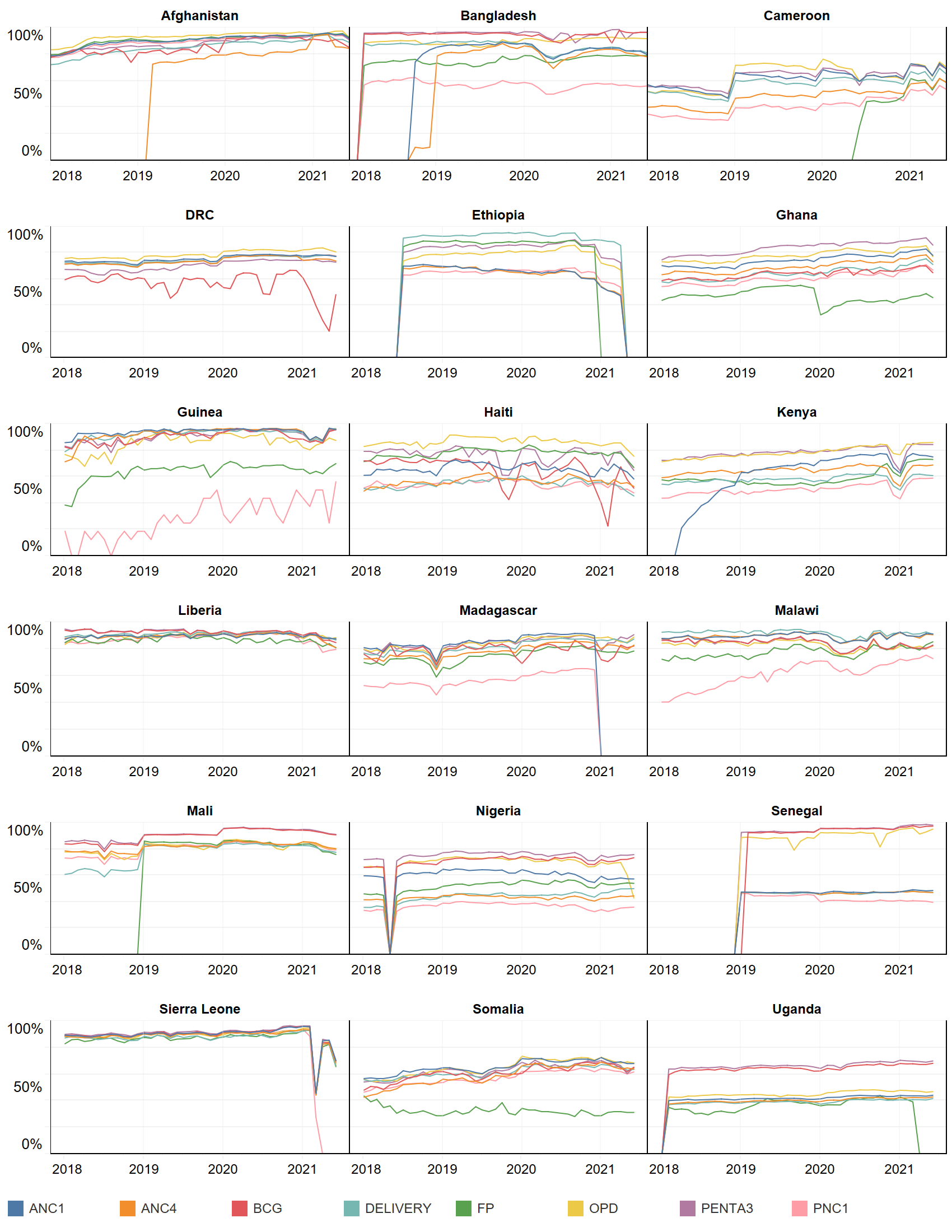


**Note:** Monthly completeness is defined as the proportion of facilities which have submitted a non-missing report, divided by the number of facilities that reported at least once during the calendar year. Annual shifts in completeness of multiple indicators may indicate a change in the denominator due to rollout to additional facilities or closure of defunct facilities during the year. DRC is Democratic Republic of the Congo. ANC1 refers to First Antenatal Care Visit. ANC4 refers to the Fourth Antenatal Care Visit. BCG refers to Bacillus Calmette–Guérin vaccination. FP refers to Family Planning Consultations. OPD refers to Outpatient visits. Penta3 refers to the Third dose of Pentavalent vaccine. PNC1 refers to First Postnatal Care Visit.

**Table J.** Cumulative change in service volume during the pandemic period (March 2020-June 2021) in a balanced panel of facilities

| **Country** | **OPD** | **FP** | **ANC1** | **ANC4** | **Delivery** | **PNC1** | **BCG** | **Penta3** |
| --- | --- | --- | --- | --- | --- | --- | --- | --- |
| Afghanistan | -9.2%*** | -2.70% | -7.9%** | -2.30% | -5.1%** | -9.8%** | 6.8%** | -0.30% |
| Bangladesh | -40.2%*** | - | -32.2%*** | 4.80% | -21.4%*** | -20.0%*** | -3.6%* | -12.4%*** |
| Cameroon | -1.20% | - | 2.50% | -6.00% | -0.40% | -2.30% | - | -2.00% |
| DRC | -6.5%*** | - | 0.80% | 0.90% | -2.5%*** | -1.8%** | - | -0.40% |
| Ethiopia | -17.1%*** | 1.00% | -3.7%*** | -2.7%** | 2.3%** | 0.80% | - | -2.6%*** |
| Ghana | -18.5%*** | - | 3.2%*** | -0.40% | -1.8%** | 1.30% | -6.0%*** | 0.20% |
| Guinea | -14.70% | 4.10% | -0.40% | -6.7%* | -3.00% | - | -0.30% | -1.40% |
| Haiti | -22.8%*** | 10.1%* | -6.7%* | -10.1%** | -25.7%*** | -15.9%*** | -27.50% | -2.30% |
| Kenya | -27.6%*** | -0.10% | -5.2%*** | -11.1%*** | -8.7%** | -7.50% | - | -2.3%** |
| Liberia | 3.80% | 6.90% | -2.70% | -6.20% | -1.70% | -6.6%** | 4.7%** | -3.00% |
| Madagascar | -8.6%*** | -1.50% | -1.00% | -1.60% | 2.10% | -3.50% | 3.40% | -0.10% |
| Malawi | -10.1%** | 6.80% | 5.60% | 2.10% | -3.80% | 2.90% | 3.70% | -6.30% |
| Mali | -6.5%** | -10.4%* |  | - | -4.4%* | -1.40% | -9.9%*** | -13.2%*** |
| Nigeria | -13.3%*** | -4.70% | -6.6%*** | -20.2%*** | -14.6%** | -1.20% | 0.90% | -1.40% |
| Senegal | -11.2%*** | - | -5.40% | -7.60% | 3.80% | -1.30% | 4.8%** | -8.6%*** |
| Sierra Leone | -13.5%*** | -21.9%** | -1.20% | 6.7%** | -3.9%** | -4.3%** | -4.7%** | -7.4%** |
| Somalia | 5.10% | - | -1.20% | 6.20% | -1.20% | 3.50% | 3.80% | 0.70% |
| Balanced panel average | -12.48% | -1.13% | -3.88% | -3.39% | -5.29% | -4.19% | -1.84% | -3.69% |
| Unbalanced panel average | -13.1% | -4.4% | -4.1% | -4.6% | -3.7% | -2.6% | -3.0% | -4.1% |

Note: The balanced panel is comprised of the subset of facilities which had no non-missing reports for the months included in the analysis for each indicator between January 2018 to June 2021. Uganda is omitted from this test as there is no consistent identifier across the entire time period. DRC is Democratic Republic of the Congo. ANC1 refers to First Antenatal Care Visit. ANC4 refers to the Fourth Antenatal Care Visit. BCG refers to Bacillus Calmette–Guérin vaccination. FP refers to Family Planning Consultations. OPD refers to Outpatient visits. Penta3 refers to the Third dose of Pentavalent vaccine. PNC1 refers to First Postnatal Care Visit.

**Table K. Sample size for balanced panel analysis (number of facilities)**

|  | ANC1 | ANC4 | BCG | DELIVERY | FP | OPD | PENTA3 | PNC1 | Grand Total |
| --- | --- | --- | --- | --- | --- | --- | --- | --- | --- |
| Afghanistan | 1,088 | 801 | 584 | 883 | 1,135 | 1,544 | 983 | 1,011 | 1,869 |
| Bangladesh | 309 | 26 | 367 | 461 |  | 5,377 | 414 | 1,021 | 6,814 |
| Cameroon | 560 | 226 |  | 364 | 1 | 250 | 382 | 62 | 916 |
| DRC | 4,915 | 4,149 | 81 | 4,306 |  | 4,957 | 1,595 | 3,759 | 7,034 |
| Ethiopia | 3,289 | 2,664 |  | 2,125 | 8,040 | 3,415 | 6,299 | 2,245 | 10,419 |
| Ghana | 1,634 | 1,000 | 643 | 783 | 61 | 1,426 | 2,793 | 709 | 3,979 |
| Guinea | 220 | 215 | 101 | 147 | 11 | 14 | 89 | 2 | 312 |
| Haiti | 88 | 55 | 5 | 59 | 101 | 179 | 41 | 44 | 278 |
| Kenya | 946 | 740 |  | 832 | 359 | 2,139 | 1,798 | 181 | 3,583 |
| Liberia | 345 | 309 | 297 | 357 | 179 | 260 | 310 | 268 | 419 |
| Madagascar | 1,158 | 532 | 318 | 831 | 384 | 1,033 | 620 | 167 | 1,454 |
| Malawi | 199 | 202 | 79 | 174 | 31 | 68 | 73 | 10 | 342 |
| Mali |  |  | 758 | 486 | 672 | 724 | 909 | 585 | 1,176 |
| Nigeria | 2,615 | 647 | 4,048 | 788 | 1,066 | 4,074 | 5,650 | 358 | 7,964 |
| Senegal | 1,137 | 1,092 | 1,270 | 914 |  | 1,026 | 1,330 | 322 | 1,563 |
| Sierra Leone | 628 | 496 | 514 | 360 |  | 432 | 612 | 688 | 918 |
| Somalia | 124 | 55 | 60 | 97 | 4 | 109 | 90 | 52 | 174 |
| Grand Total | 20,757 | 15,228 | 10,124 | 15,105 | 14,213 | 28,789 | 25,237 | 11,484 | 51,516 |

Note: DRC is Democratic Republic of the Congo. ANC1 refers to First Antenatal Care Visit. ANC4 refers to the Fourth Antenatal Care Visit. BCG refers to Bacillus Calmette–Guérin vaccination. FP refers to Family Planning Consultations. OPD refers to Outpatient visits. Penta3 refers to the Third dose of Pentavalent vaccine. PNC1 refers to First Postnatal Care Visit.

**Table L. Data considerations**

| Afghanistan | ANC4 data unavailable before March 2019. |
| --- | --- |
| Bangladesh | Lower-level and upper-level facilities are in different systems. There is no information on ANC care in lower-level facilities, ANC data in upper-level facilities is available starting October 2018. January and February 2018 dropped due to poor completeness tied to elections. No relevant family planning data or close proxy was available. |
| Cameroon | Family planning and BCG data were dropped due to low completeness. |
| DRC | Family planning data was dropped due to low completeness. |
| Ethiopia | BCG data was dropped due to low completeness. Data before July 2018 (start of Ethiopian liturgical year) is poor completeness, as the HMIS system was rolling out. Data were unavailable past April 2021 at the time of analysis, and in 2021 for family planning. Data from Tigray is unavailable due to security conditions. |
| Ghana | No omissions |
| Guinea | Guinea HMIS has data only from hospitals and health centers, lower-level facilities are not reported. Data are only available start March 2018 for ANC4, and May 2018 for family planning. PNC data are omitted for poor completeness. |
| Haiti | No data on BCG. Data past May 2021 were poor completeness at the time of analysis |
| Kenya | No data for BCG available. ANC1 data is available starting July 2018 |
| Liberia | No omissions |
| Madagascar | There is no updated data for ANC1 and PNC1 in 2021 at the time of analysis. No data from lower-level facilities was reported. |
| Malawi | No omissions |
| Mali | Antenatal care is measured in aggregate, no close ANC1 or ANC4 proxy was identified. Family planning indicators were dropped for poor quality in 2018. There is limited reporting from hospitals. |
| Nigeria | Data from June 2021 were dropped for low completeness at the time of analysis. A change in indicator definitions occurred in October 2020 |
| Senegal | Data from 2018 were not available at the time of analysis. Family planning indicators were dropped for poor completeness. |
| Sierra Leone | System issues resulted impacted reporting in March 2021 and June 2021. PNC data are not available after February 2021 at time of analysis. |
| Somalia | The state of Somaliland is not included. Family planning data are not available. |
| Uganda | System change in 2020 resulted in indicator definition changes and inability to track individual health facilities. High completeness family planning and PNC1 indicators were not identified. |

Note: DRC is Democratic Republic of the Congo. ANC1 refers to First Antenatal Care Visit. ANC4 refers to the Fourth Antenatal Care Visit. BCG refers to Bacillus Calmette–Guérin vaccination. FP refers to Family Planning Consultations. OPD refers to Outpatient visits. Penta3 refers to the Third dose of Pentavalent vaccine. PNC1 refers to First Postnatal Care Visit.

**Table M.** Percentage of reporting outliers by country in the pre-pandemic (January 2018 – February 2020) and the pandemic (March 2020 – June 2021) periods

| Country | Time period | ANC1 | ANC4 | BCG | DELIVERY | FP | OPD | PENTA3 | PNC1 |
| --- | --- | --- | --- | --- | --- | --- | --- | --- | --- |
| Afghanistan | Pre-pandemic | 0.30% | 0.00% | 0.30% | 0.00% | 0.70% | 0.40% | 0.50% | 0.10% |
| Afghanistan | Pandemic | 0.50% | 0.50% | 0.40% | 0.60% | 0.80% | 0.60% | 0.70% | 0.40% |
| Bangladesh | Pre-pandemic | 1.90% | 0.60% | 0.40% | 0.20% |  | 0.20% | 0.10% | 0.00% |
| Bangladesh | Pandemic | 0.80% | 0.20% | 2.10% | 0.30% |  | 0.10% | 2.00% | 0.00% |
| Cameroon | Pre-pandemic | 0.10% | 0.00% |  | 0.00% |  | 0.30% | 0.10% | 0.00% |
| Cameroon | Pandemic | 0.10% | 0.10% |  | 0.00% |  | 0.40% | 0.10% | 0.00% |
| DRC | Pre-pandemic | 1.10% | 1.20% | 0.20% | 0.80% |  | 1.10% | 0.50% | 0.90% |
| DRC | Pandemic | 1.00% | 1.10% | 0.50% | 0.90% |  | 1.60% | 1.40% | 0.70% |
| Ethiopia | Pre-pandemic | 0.10% | 0.10% |  | 0.10% | 0.70% | 1.10% | 0.00% | 0.10% |
| Ethiopia | Pandemic | 0.10% | 0.10% |  | 0.20% | 0.90% | 1.80% | 0.00% | 0.10% |
| Ghana | Pre-pandemic | 0.00% | 0.10% | 0.00% | 0.00% | 0.20% | 0.40% | 0.00% | 0.00% |
| Ghana | Pandemic | 0.00% | 0.10% | 0.00% | 0.00% | 0.20% | 0.40% | 0.00% | 0.00% |
| Guinea | Pre-pandemic | 0.70% | 0.40% | 1.20% | 0.20% | 2.00% | 1.00% | 1.20% | 1.90% |
| Guinea | Pandemic | 0.80% | 0.40% | 1.00% | 0.10% | 1.00% | 0.60% | 0.50% | 1.20% |
| Haiti | Pre-pandemic | 0.00% | 0.00% | 0.30% | 0.10% | 1.50% | 1.50% | 0.30% | 0.10% |
| Haiti | Pandemic | 0.00% | 0.10% | 0.20% | 0.00% | 1.50% | 1.00% | 0.30% | 0.00% |
| Kenya | Pre-pandemic | 0.00% | 0.00% |  | 0.00% | 0.70% | 0.90% | 0.00% | 0.10% |
| Kenya | Pandemic | 0.10% | 0.00% |  | 0.10% | 0.80% | 0.70% | 0.10% | 0.10% |
| Liberia | Pre-pandemic | 1.00% | 2.00% | 1.20% | 1.00% | 2.80% | 0.90% | 0.70% | 3.00% |
| Liberia | Pandemic | 0.70% | 1.20% | 0.30% | 0.60% | 2.80% | 0.60% | 0.10% | 1.60% |
| Madagascar | Pre-pandemic | 0.10% | 0.00% | 0.30% | 0.00% | 0.30% | 0.60% | 0.30% | 0.00% |
| Madagascar | Pandemic | 0.10% | 0.00% | 0.20% | 0.00% | 0.40% | 1.00% | 0.10% | 0.00% |
| Malawi | Pre-pandemic | 1.10% | 1.00% | 1.30% | 0.60% | 1.30% | 1.00% | 1.20% | 0.10% |
| Malawi | Pandemic | 1.30% | 1.30% | 1.00% | 0.70% | 1.40% | 1.00% | 1.00% | 0.10% |
| Mali | Pre-pandemic |  |  | 0.60% | 1.80% | 1.90% | 0.60% | 0.60% | 1.20% |
| Mali | Pandemic |  |  | 1.30% | 3.10% | 1.80% | 0.80% | 1.00% | 1.70% |
| Nigeria | Pre-pandemic | 0.20% | 0.20% | 0.10% | 0.00% | 0.40% | 0.70% | 0.10% | 0.10% |
| Nigeria | Pandemic | 0.20% | 0.10% | 0.10% | 0.00% | 0.50% | 0.50% | 0.10% | 0.20% |
| Senegal | Pre-pandemic | 0.10% | 0.20% | 1.00% | 0.40% |  | 1.20% | 0.40% | 0.30% |
| Senegal | Pandemic | 0.00% | 0.00% | 0.50% | 0.00% |  | 1.30% | 0.40% | 0.10% |
| Sierra Leone | Pre-pandemic | 0.10% | 0.00% | 0.00% | 0.00% | 0.80% | 0.70% | 0.00% | 0.00% |
| Sierra Leone | Pandemic | 0.00% | 0.20% | 0.00% | 0.00% | 1.10% | 0.70% | 0.00% | 0.10% |
| Somalia | Pre-pandemic | 0.60% | 0.40% | 0.60% | 0.10% |  | 1.00% | 0.70% | 0.50% |
| Somalia | Pandemic | 0.30% | 0.20% | 0.40% | 0.10% |  | 0.60% | 0.30% | 0.20% |
| Uganda | Pre-pandemic | 0.10% | 0.10% | 0.50% | 0.40% |  | 0.50% | 0.30% |  |
| Uganda | Pandemic | 0.30% | 0.30% | 0.40% | 0.40% |  | 0.60% | 0.30% |  |

Note: DRC is Democratic Republic of the Congo. ANC1 refers to First Antenatal Care Visit. ANC4 refers to the Fourth Antenatal Care Visit. BCG refers to Bacillus Calmette–Guérin vaccination. FP refers to Family Planning Consultations. OPD refers to Outpatient visits. Penta3 refers to the Third dose of Pentavalent vaccine. PNC1 refers to First Postnatal Care Visit.

**Table O.** Population totals and mortality rates references for analyzed countries

| **Country** | **Population, total** | **Mortality rate, under-5 (per 1,000 live births)** | **Maternal mortality ratio (national estimate, per 100,000 live births)** | **Maternal mortality ratio (modeled estimate, per 100,000 live births)** |
| --- | --- | --- | --- | --- |
| Afghanistan | 38928341 | 58 | 1194 | 638 |
| Bangladesh | 164689383 | 29.1 | 215 | 173 |
| Cameroon | 26545864 | 72.2 |  | 529 |
| Congo, Democratic Republic | 89561404 | 81.2 |  | 473 |
| Ethiopia | 114963583 | 48.7 | 557 | 401 |
| Ghana | 31072945 | 44.7 | 334 | 308 |
| Guinea | 13132792 | 95.6 | 833 | 576 |
| Haiti | 11402533 | 60.5 | 732 | 480 |
| Kenya | 53771300 | 41.9 |  | 342 |
| Liberia | 5057677 | 78.3 |  | 661 |
| Madagascar | 27691019 | 50.2 |  | 335 |
| Malawi | 19129955 | 38.6 | 451 | 349 |
| Mali | 20250834 | 91 |  | 562 |
| Nigeria | 206139587 | 113.8 |  | 917 |
| Senegal | 16743930 | 38.1 | 440 | 315 |
| Sierra Leone | 7976985 | 107.8 |  | 1120 |
| Somalia | 15893219 | 114.6 |  | 829 |
| Uganda | 45741000 | 43.3 | 481 | 375 |

Note: Reference data from World Bank World Development Indicator (<https://databank.worldbank.org/>). Accessed May 13, 2022.

**Fig B.** Estimated and observed volume of additional indicators with officially reported COVID-19 deaths per 100,000 and mobility restrictions by country, January 2018 -June 2021


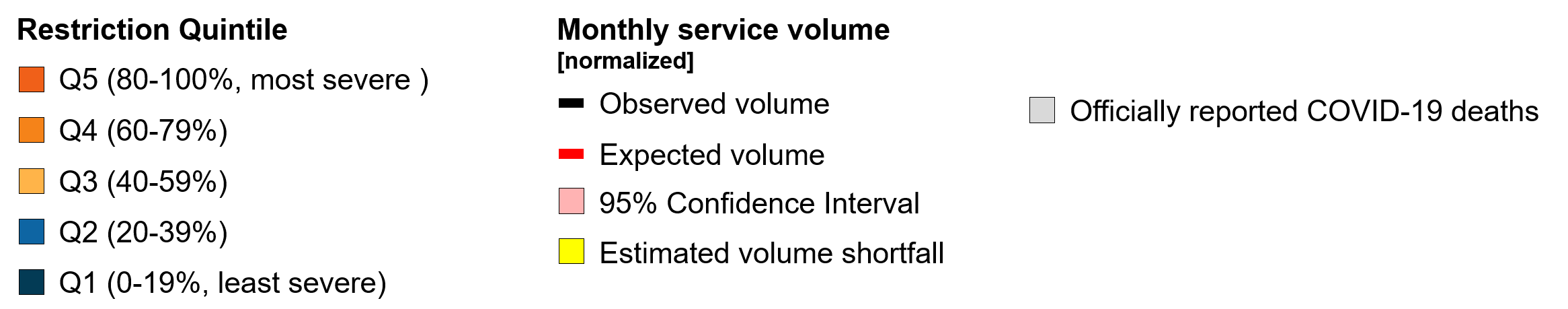


1. ANC1


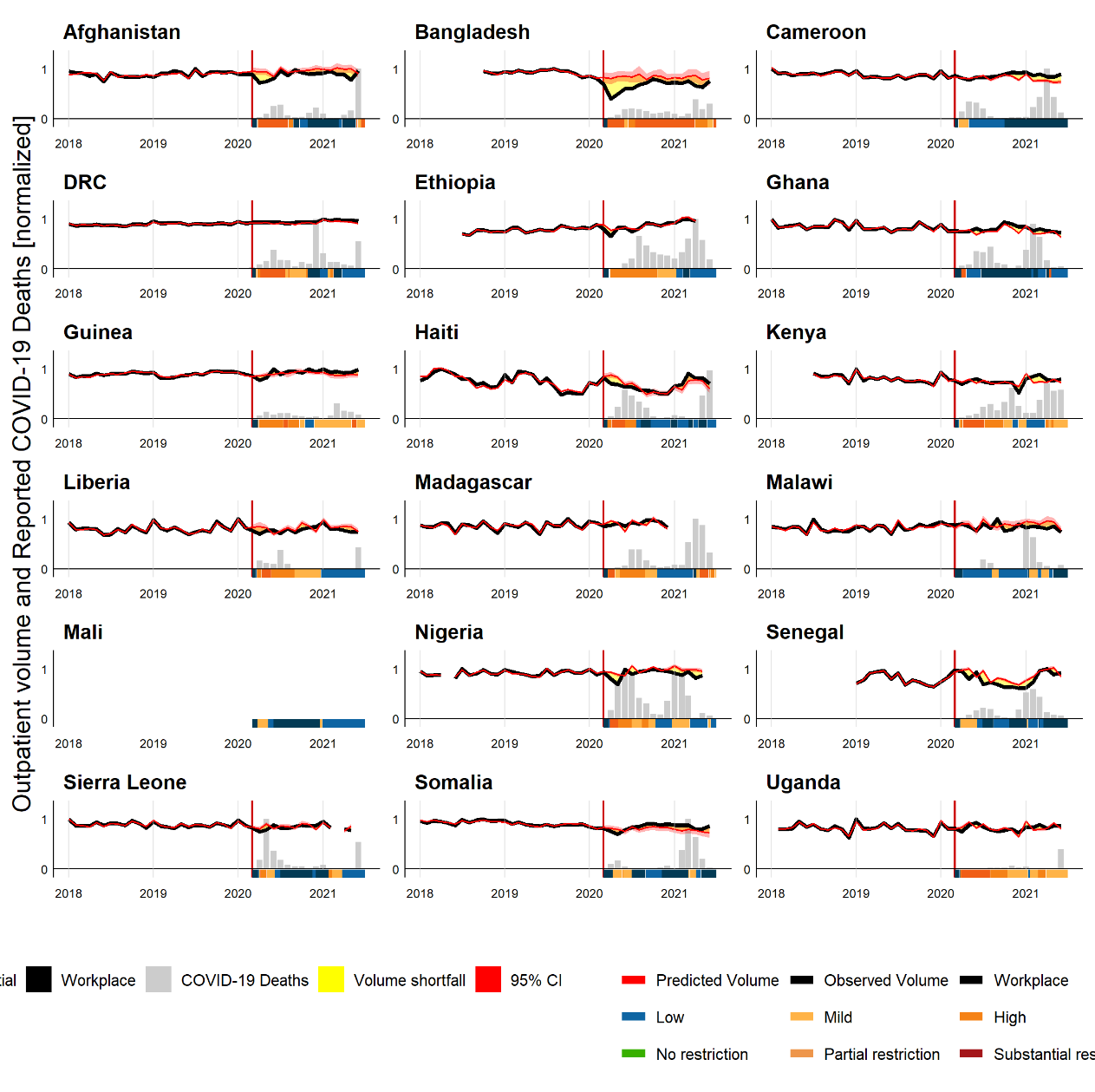


1. Delivery


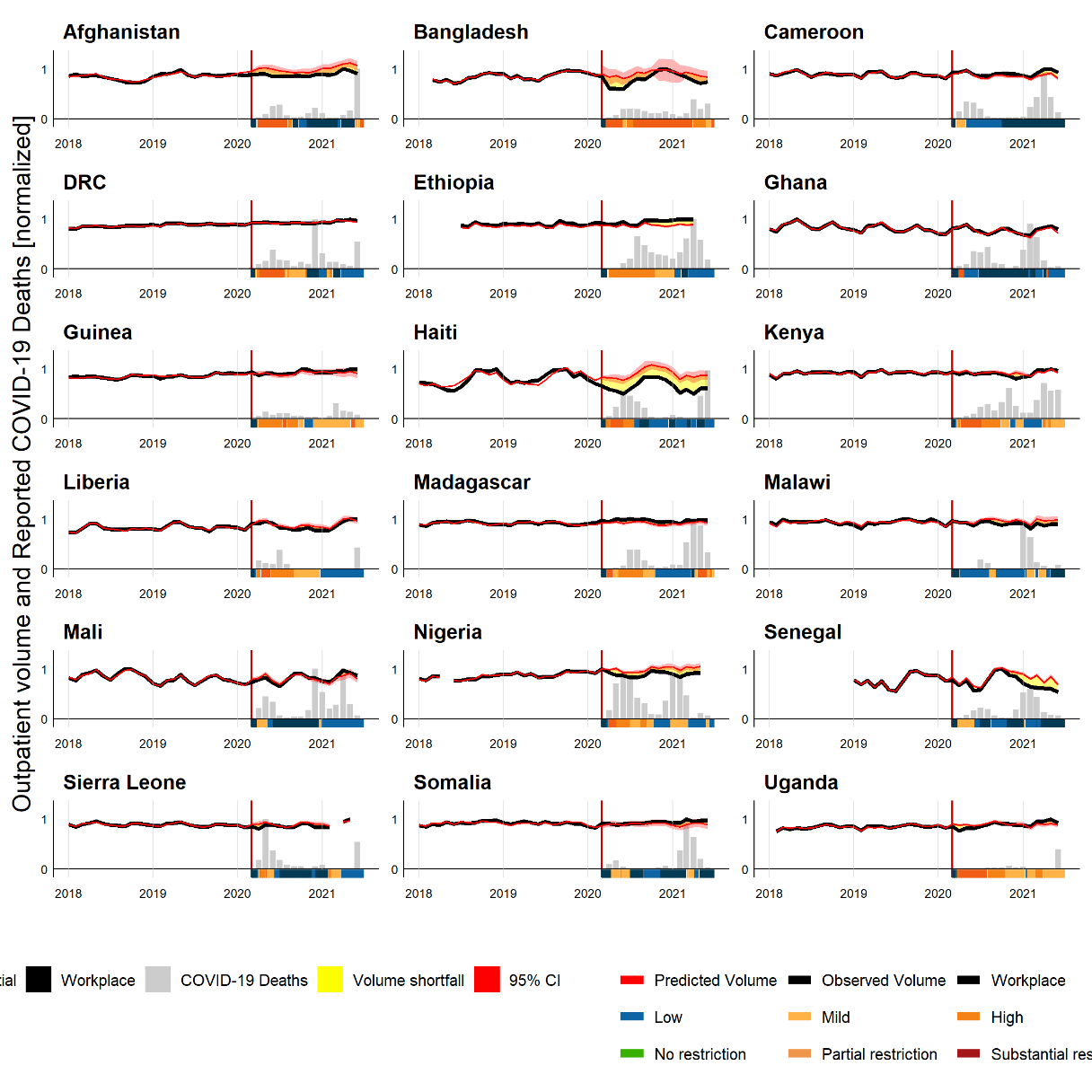


1. BCG


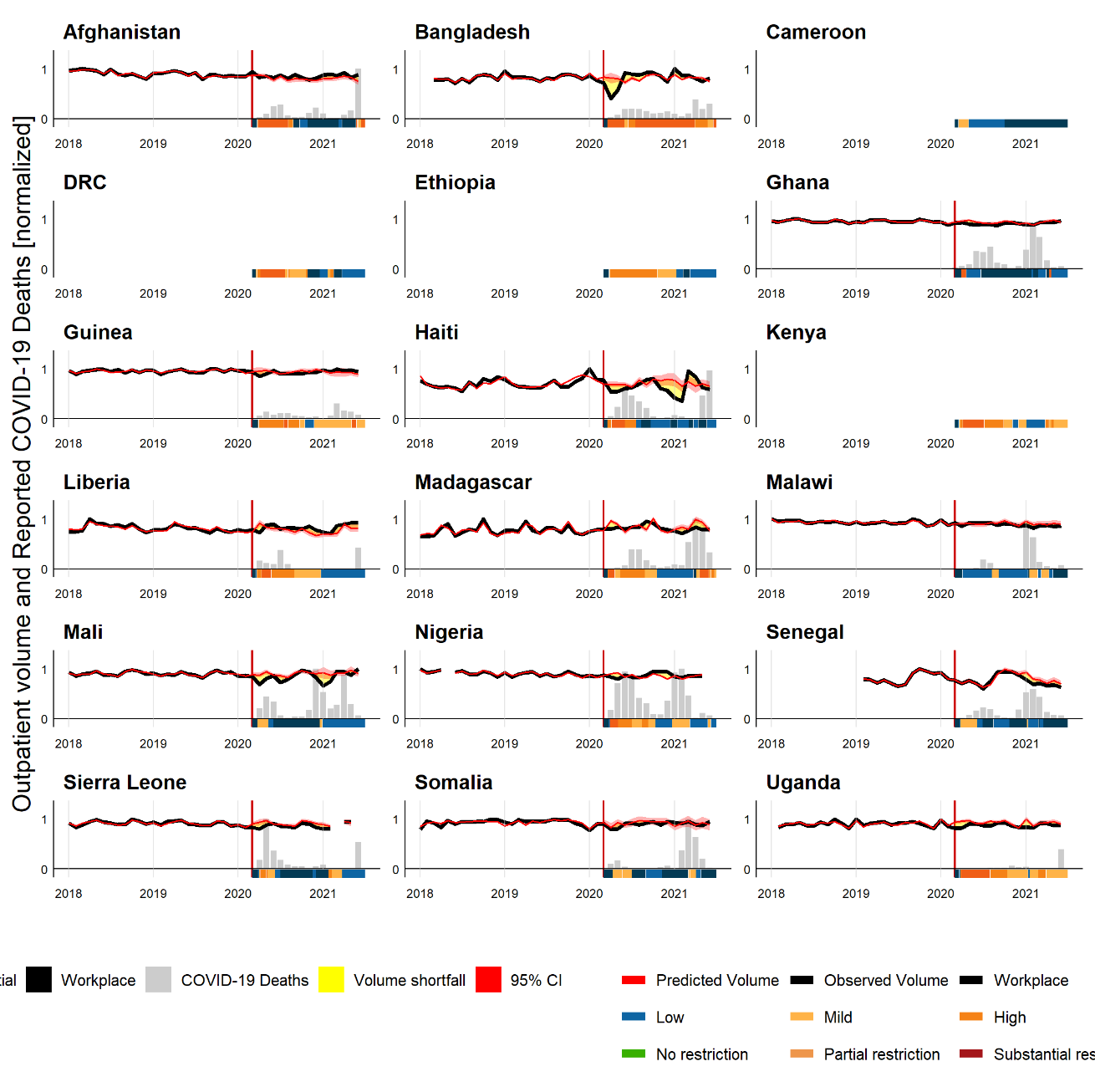


1. Penta3


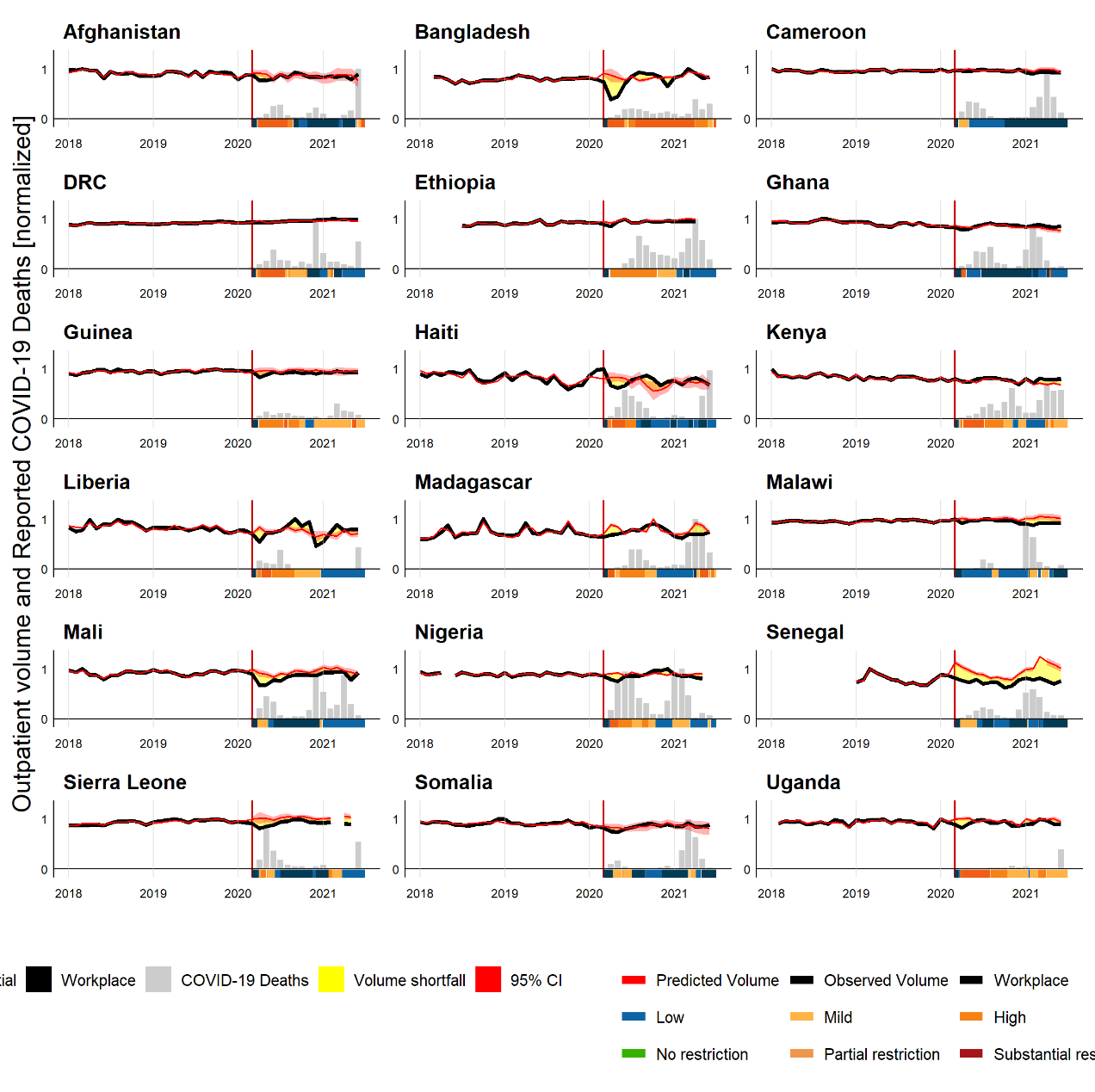

Supplement: S1 Text — Table A. HMIS indicator definition and mapping. Table B. Sensitivity of disruption estimates between alternative definitions for deliveries. Table C. Sensitivity of disruption estimates between alternative definitions for outpatient consultations. Table D. Sensitivity of disruption estimates between alternative definitions for family planning. Table E. Linkage between service indicators to LiST interventions. Table F. Difference between expected and observed service coverage by month and country. Table G. Projections of mortality from LiST Model by Quarter. Table H. Bounding the mortality estimates using service disruption confidence intervals. Table I. Sensitivity analysis of linking decisions. Text A. Data notes. Fig A. Level of completeness by country and indicator. Table J. Cumulative change in service volume during the pandemic period (March 2020–June 2021) in a balanced panel of facilities. Table K. Sample size for balanced panel analysis (number of facilities). Table L. Data considerations. Table M. Percentage of reporting outliers by country in the prepandemic (January 2018–February 2020) and the pandemic (March 2020–June 2021) periods. Table N. Population totals and mortality rates references for analyzed countries. Fig B. Estimated and observed volume of additional indicators with officially reported COVID-19 deaths per 100,000 and mobility restrictions by country, January 2018–June 2021. (DOCX) [file pmed.1004070.s002.docx]
